# Artificial intelligence diet plans underestimate nutrient intake compared to dietitians in adolescents

**DOI:** 10.3389/fnut.2026.1765598

**Published:** 2026-03-12

**Authors:** Ayşe Betül Bilen, Gülen Ecem Kalkan, Hülya Yılmaz Önal

**Affiliations:** 1 Department of Nutrition and Dietetics, Faculty of Health Sciences, Istanbul Atlas University, Istanbul, Türkiye; 2 Department of Nutrition and Dietetics, Faculty of Health Sciences, Istanbul Medeniyet University, Istanbul, Türkiye

**Keywords:** adolescent nutrition, artificial intelligence, diet planning, large language models, nutrient adequacy

## Abstract

**Objective:**

Although artificial intelligence (AI)-based nutrition recommendations are becoming increasingly common among the public, the accuracy and reliability of diets produced especially for adolescents in the growth and development period are not sufficiently known. This study aimed to evaluate the clinical validity of AI by comparing the nutritional content of diets generated by different AI models with dietitian reference plans.

**Methods:**

A total of 60 three-day diet plans were generated in two sessions by five AI models (ChatGPT-4o, Gemini 2.5 Pro, Claude 4.1, Bing Chat-5GPT, and Perplexity) for four standardized adolescent profiles in this cross-sectional and comparative study. A dietitian reference plan was prepared for each profile. Energy and macro-micronutrients were analyzed with BeBiS. Comparisons were evaluated with single-sample *t*-test, Cohen’s d, and Bland–Altman fit analyses.

**Results:**

AI models tended to systematically undercalculate energy (bias: +695 kcal), protein (+19.9 g), lipid (+15.8 g), and carbohydrate (+114.6 g). In macronutrient percentages, protein (21.5–23.7%) and lipid (41.5–44.5%) ratios were above the recommended adolescent guidelines, while carbohydrate ratios (32.4–36.3%) were significantly below. Significant variation was observed between models in micronutrient contents, and no model showed consistent proximity to the dietitian across all nutrients.

**Conclusion:**

AI models have exhibited clinically significant deviations in diet plans for adolescents at both macro and micro levels. The findings indicate that AI-based dietary recommendations are not appropriate to use without professional supervision, emphasizing the need for model improvements for more reliable data generation in this area.

## Graphical abstract

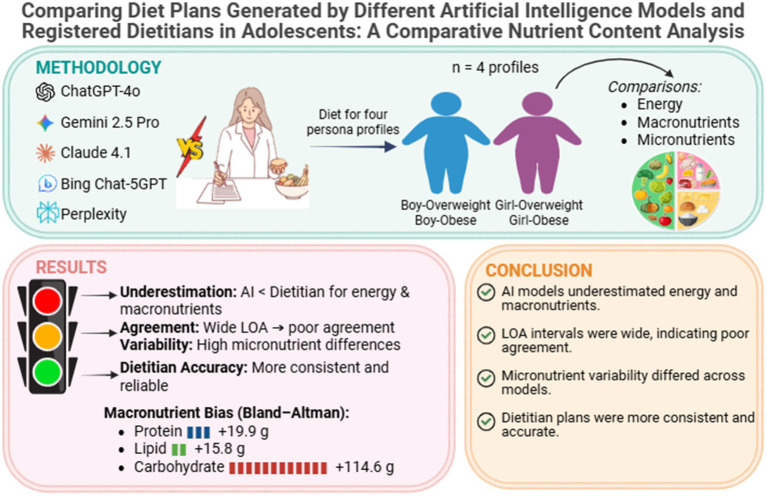

## 1 Introduction

Adolescent overweight and obesity are rapidly increasing global public health problems in both developed and developing countries (1–3). According to the World Health Organization (WHO), the number of overweight children and adolescents aged 5–19 years reached approximately 390 million in 2022, of whom 160 million were classified as obese (1). UNICEF reports indicate that, in many regions, overweight in the 5–19 age group has become the predominant form of malnutrition, shifting the traditional picture of undernutrition in childhood and adolescence (2). Adolescent obesity is associated with cardiometabolic problems such as type 2 diabetes, dyslipidemia, hypertension, non-alcoholic fatty liver disease, and sleep apnea, as well as reduced quality of life and an increased risk of obesity in adulthood (3–5). For this reason, preventing obesity during adolescence and treating existing obesity early and effectively is critical for both short- and long-term health outcomes (6).

Current guidelines recommend multidisciplinary, child-focused and family-centered long-term programs that combine medical nutrition therapy, increased physical activity and behavioral modification as the cornerstone of obesity management (7–9). National and international guidance highlights that intensive lifestyle modification programs should form the core of treatment, and that individualized medical nutrition therapy (MNT) targeting energy balance, macro- and micronutrient distribution and meal patterns should be central to these interventions (7–9). Within this framework, dietitians are key health professionals who design and monitor guideline-based, sustainable and individualized nutrition plans, taking into account growth and developmental needs, comorbidities, family and school food environments, socioeconomic status and cultural eating habits (7–9). However, in many countries, limited access to dietitians and high clinical workloads make it difficult for adolescents to receive regular, personalized nutrition counseling (3).

Digital health applications and artificial intelligence (AI)-based tools are increasingly being explored as ways to alleviate these constraints. A review of chatbots designed to support nutrition and physical activity in adolescents identified only five relevant studies; across these interventions, chatbot use and acceptability were generally moderate to high, but findings on health-related outcomes such as weight and behavior change were few and heterogeneous, and the overall level of evidence was judged to be limited (10). In a survey of 315 high school students aged 15–19 years in Bulgaria, 31.4% of participants reported using the internet (including ChatGPT) to obtain information about healthy eating, whereas only 10.5% had consulted a family doctor for this purpose (11). In the same study, 18.1% of students reported body dissatisfaction, 24.8% a desire to be thinner, and 10.5% reported unhealthy weight control behaviors such as post-meal vomiting or laxative use (11). Taken together, these findings suggest that a notable proportion of adolescents with pronounced body image and weight concerns rely on internet- and AI-based sources for nutrition information, and that the quality and safety of information obtained from these tools may be clinically important (10, 11).

In parallel, ChatGPT and other large language models (LLMs) have begun to be evaluated in the nutrition field for answering dietary questions, generating educational content and creating sample menu plans (12–15). In a study among university students, ChatGPT’s responses to nutrition knowledge questions were found to be approximately 84.4% accurate according to a nutrition literacy test; however, dietitians judged these responses to be limited in terms of understandability, practical applicability and breadth of coverage (12). In another study comparing diets generated by ChatGPT with diets planned by dietitians for chronic disease scenarios, ChatGPT-generated menus did not consistently meet energy and nutrient targets, sometimes included foods that could be clinically inappropriate, and showed a low level of individualization (13). A study comparing nutrition recommendations from several chatbots (ChatGPT, Gemini, Copilot, Claude, Perplexity and others) reported that accuracy, completeness and consistency scores were particularly low in cases with comorbidities and concluded that these tools cannot replace the services of a registered dietitian (15). Similarly, a qualitative study underlined that while ChatGPT may support dietitians with basic nutrition information and literature searches, it cannot assume core professional roles such as patient-specific medical nutrition therapy planning, ethical responsibility and long-term follow-up, and therefore cannot substitute for professional nutrition counseling (14).

Recently, not only ChatGPT but also other large language models such as Gemini, Claude and Microsoft Copilot have begun to be evaluated for their ability to generate nutrition and dietary recommendations. Several studies have compared the responses of multiple chatbots for the same clinical nutrition scenario. Overall, these models appear to broadly reflect guideline principles; however, in more complex clinical cases, their accuracy and consistency remain limited, and contradictory or potentially clinically harmful deviations in nutrient recommendations—particularly for protein—have been reported (15). In another study, weight-loss diet plans ranging from 1,400 to 1800 kcal generated by ChatGPT-4, Gemini and Copilot were compared. Although all chatbots produced plans with generally acceptable diet quality, there were noticeable deviations from the intended energy targets and marked differences in macronutrient and fatty acid distribution (16). A systematic review summarizing ChatGPT’s performance in meal planning and dietary recommendations found that most studies reported overall satisfactory accuracy, and in some instances results comparable to, or even better than, those of human dietitians. At the same time, the review emphasized important limitations regarding safety, nutrient adequacy, adaptation to specific clinical conditions and translation into real-world practice (17). Taken together, these findings suggest that different LLMs may provide accessible and relatively strong informational support in nutrition but still show clear limitations when it comes to generating safe and nutritionally adequate diet plans (12–17).

However, most of this evidence comes from adult populations and from hypothetical or scenario-based clinical cases. According to current systematic reviews and recent literature, there are no studies that directly compare diets generated by AI-based systems for overweight and obese adolescents with individualized medical nutrition therapy planned by a dietitian for the same individual, in terms of energy and nutrient content, safety and feasibility (12, 13, 17). In addition, only a small number of studies have examined how much diets produced by different chatbots (e.g., LLMs such as ChatGPT, Gemini and Microsoft Copilot) for the same clinical scenario diverge from one another and from a dietitian-planned diet. Existing findings indicate that there can be meaningful differences in energy and nutrient levels—and at times clinically important deviations—between the diets recommended by these models (15–17).

The present study aims to compare guideline-based, individualized diets planned by a dietitian for overweight and obese adolescents with diets generated for the same individuals by multiple AI-based chatbots (ChatGPT, Gemini, Claude, Microsoft Copilot/Bing Chat and Perplexity). Specifically, it will examine the extent to which the total energy, macronutrient and selected micronutrient content of diets produced by each AI model deviate from the dietitian-prepared diet. In doing so, the study seeks to provide evidence on whether commonly used AI-based chatbots could replace dietitian-centered care in overweight and obese adolescents, or whether they should instead be regarded as complementary tools to be used only under dietitian supervision.

## 2 Materials and methods

### 2.1 Study population and design

**Figure 1:**
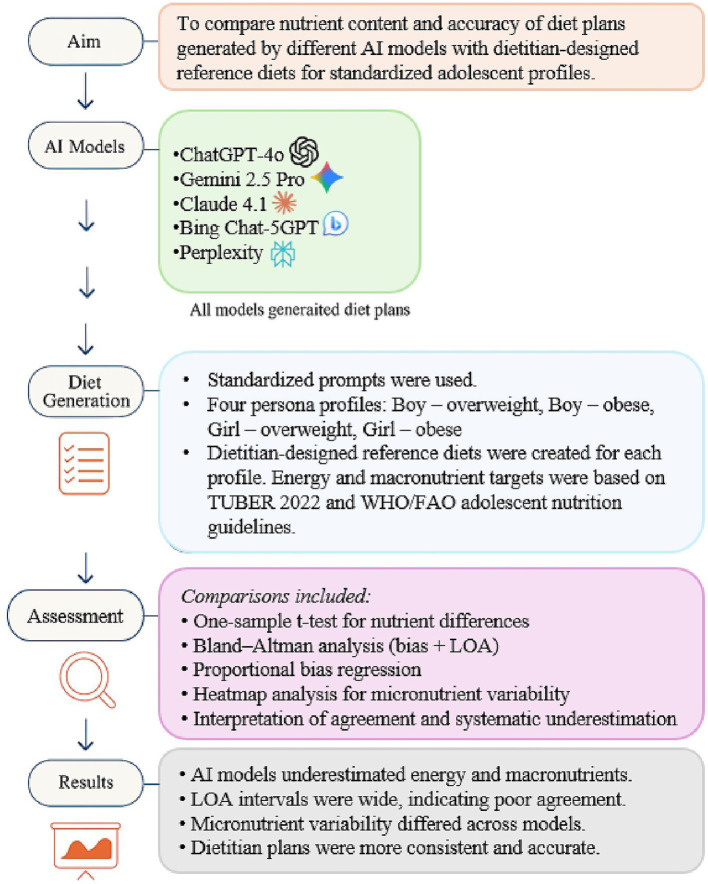
Methodological framework for comparing AI-generated diet plans with dietitian-designed reference diets for adolescents. Five artificial intelligence models produced three-day diet plans for four standardized adolescent profiles using uniform prompts (60 AI-generated diet plans evaluated against 4 dietitian-designed reference diets). All plans were analyzed for nutrient content, and comparisons included one-sample *t*-tests for energy and macronutrients, Bland–Altman analysis to assess method agreement, proportional bias regression, and heatmap visualization for micronutrient variability.

As shown in Figure 1, this study used a multi-layered methodological framework that includes the creation of AI model outputs, reference diets prepared by the dietitian, standard adolescent profiles, and multifaceted complementary analyses (single-sample *t*-tests, Bland–Altman analysis, proportional deviation regression, and micronutrient heatmaps). The following subsections describe these components in detail.

### 2.2 Artificial intelligence systems and prompting protocol

The study was designed to be based on the multi-prompt × evaluator model and adapted based on approaches in the AI-nutrition literature (18). The study used five different AI models: ChatGPT-4o (OpenAI) (19), Gemini 2.5 Pro (Google DeepMind) (20), Bing Chat-5GPT (Microsoft) (21), Claude 4.1 (Anthropic) (22), and Perplexity (Perplexity AI) (23). These chatbots are chosen because they are widely used AI tools that can generate personalized diet plans. Free versions of AI tools have been preferred because they are a more accessible tool for the adolescent population compared to paid versions. A new email account was created and used when logging into each chatbot to minimize the impact of previous user interactions; Thus, the responses of artificial intelligences are prevented from being affected by previous learning. Each model was run in two separate sessions for four different adolescent profiles. Thus, a total of 60 diet plans (4 adolescent profiles x 5 artificial intelligence applications x 3-day diet plans) were obtained, each for 3 days. This design was chosen to evaluate both intersession reproducibility and intra-model consistency. The three-day plans of each model were compared with the reference diet plans prepared by the dietitian in accordance with the guidelines and guides.

### 2.3 Adolescent profiles scenarios

In the study, four hypothetical adolescent profiles standardized in terms of age, gender and body mass index (BMI) were created. In the study, 15 years was chosen for the adolescent profile. The reason for this choice is that the WHO defines adolescence in the age range of 10–19 years and the age of 15 is in the middle of this range (24). Since the growth rate and nutrient requirements of girls and boys are similar at this age, gender-related developmental differences are minimized. In determining body weights, not only BMI value, but also BMI-percentile classifications were evaluated based on the CDC Extended BMI-for-Age Growth Charts specific to age and gender (25). According to this classification, the 85–94th percentile is “overweight,” ≥95. percentile is defined as “obese.” This approach is in line with the AAP and CDC’s pediatric weight management guidelines and ensures that profile categories are established with a clinically valid reference for growth and development (7, 25). Height lengths were determined according to the 50th percentile value according to age; The weights were chosen to represent the slightly overweight and obese categories in girls and boys. The four profiles created accordingly are as follows:

- Boy – Overweight (85–94th percentile): 170 cm, 73 kg (BMI 25.3 kg/m^2^).
- Boy – Obese (≥95th percentile): 170 cm, 89 kg (BMI 30.8 kg/m^2^).
- Girl – Overweight (85–94th percentile): 162 cm, 67 kg (BMI 25.5 kg/m^2^).
- Girl – Obese (≥95th percentile): 162 cm, 80 kg (BMI 30.5 kg/m^2^).

### 2.4 Prompt texts used

For each artificial intelligence model, prompt texts were standardized in Turkish and the same content was used. The goal is to assess the models’ capacity to produce safe, guideline-compliant, and content-consistent dietary recommendations without external guidance. The prompts did not include any calorie targets, non-scientific statements such as “detox,” or professional nutritional guidance. This approach was chosen to reflect the typical communication style of a real adolescent; because a 15-year-old individual is not expected to give detailed instructions that include technical terms, macro goals, or guide statements. Therefore, the use of simple and natural user language provided a more realistic test condition that activated the models’ own internal decision-making mechanisms. Example prompt “I am a 15-year-old, 170 cm tall, 89 kg boy. Can you write me a 3-day weight loss nutrition plan? List it as breakfast, lunch, dinner and 2 snacks. Give portions in grams or ml. Use foods that are easy to find in Turkey.” All other prompts are given in the Supplementary Section 1. Standardized prompts including age, height, and body weight were deliberately used to ensure comparability across AI models and to minimize variability related to user input. These values were selected to represent clinically relevant adolescent profiles spanning both overweight and obesity (e.g., BMI at or above the 85th percentile for overweight and the 95th percentile for obesity, based on age- and sex-specific percentiles), consistent with the clinical context of diet planning. Although adolescents may not always provide complete anthropometric information in real-life settings, the use of structured prompts allows objective evaluation of model performance under controlled conditions. In addition, three-day diet plans were selected as they are commonly used in nutritional assessment studies and allow evaluation of day-to-day consistency while minimizing excessive repetition.

### 2.5 Dietitian-designed reference plans

The reference diets were prepared by an associate professor dietitian who specializes in pediatric and adolescent diseases, and a 1-day sample diet plan was created for each adolescent. These plans are arranged in line with the Turkish Nutrition Guidelines (26), WHO/FAO Adolescent Nutrition Guidelines (27) and Acceptable Macronutrient Distribution Ranges (AMDR) (28) defined by the Institute of Medicine (IOM). The distribution of energy to macronutrients was determined as 45–50% from carbohydrates (CHOs), 30–35% from lipid and 15–20% from protein; these ratios are fully consistent with the AMDR ranges recommended by the IOM for ages 4–18 (CHO 45–65%, lipid 25–35%, protein 10–30%). For this reason, the plans prepared by the dietitian were accepted as a clinically valid “reference method” as they holistically included the scientific principles recommended by international guidelines.

Due to the design of the study, the dietitian was not asked to create day-to-day variation. Instead, a single standardized reference menu was developed for each profile in accordance with established nutritional guidelines. Accordingly, dietitian-prepared guideline-aligned meal plans were used as a fixed reference point, while multi-day AI-generated plans were evaluated in terms of deviation, consistency, and variation relative to this reference. Dietitian-designed meal plans were used solely as reference standards and were not subject to comparative performance evaluation.

The energy requirements of adolescents were calculated by taking into account age, gender and anthropometric characteristics; Basal Metabolic Rate (BMR) was determined using Schofield’s equations (29) (BMR = 17.5 × weight (kg) + 651 in boys; BMR = 12.2 × weight (kg) + 746 in girls), Total Energy Expenditure (TEE) was calculated with the formula BMR × PAL (1.55; sedentary), and energy supplement (Eg) was added for growth (29, 30). Eg was calculated according to age-specific average daily weight gain with the formula Eg = (g/day) × 2 kcal/g; It was accepted that an average of 2 kcal/g energy was required for growth tissue synthesis (30). BMI percentile classifications were used as basis for weight management (31); Maintaining weight or slowing down the rate of increase for the BMI 85–94th percentile, ≤1 kg weight loss per week for the BMI 95–98th percentile, BMI ≥ 99. For the percentile, it is recommended to limit weight loss to ≤1 kg/week. Accordingly, energy restriction was not made in slightly obese adolescents; BMR was calculated over the 50th percentile ideal weight according to age and height, and physical activity and growth supplements were added; In obese adolescents, an average of 500 kcal of energy restriction per day was applied with a loss target of approximately 0.5 kg/week (32). In menu planning, nutritional diversity was ensured in accordance with WHO and TUBER principles; milk and its products, meat-eggs-legumes, cereals, vegetables and fruits were distributed in a balanced manner and portion sizes were arranged according to TUBER (26) portion criteria in accordance with the energy levels determined by age and gender.

### 2.6 Nutrient analysis

All AI-generated diet plans were coded and analyzed using the BeBiS (Nutrition Information System, version 9.0; Ebispro for Windows, Istanbul, Turkey) software. For each plan, energy (kcal), protein (g), lipid (g), CHO (g), and 22 micronutrients (vitamins and minerals) were calculated. When portion sizes were ambiguously described (e.g., “one bowl” or “one serving”), standardized household measures and portion definitions based on BeBiS and national dietary references were consistently applied. All AI models were instructed to generate meal plans using foods commonly available in Turkey. Accordingly, all foods and ingredients could be directly identified and coded using the BeBiS database, and no external food substitution or matching procedures were required.

### 2.7 Statistical analysis

For each adolescent profile, the dietitian prepared a single one-day reference diet in accordance with established nutritional guidelines, which served as a fixed comparison point. In contrast, each AI model generated three-day diet plans for the same profiles. To ensure comparability and reduce day-to-day variability in AI outputs, nutrient values from the three AI-generated days were averaged, and these mean values were used for all statistical comparisons with the corresponding dietitian reference diet. Accordingly, the primary unit of analysis was defined as the AI model–profile combination, rather than individual days. This approach allowed assessment of systematic deviation and agreement between AI-generated plans and guideline-based reference diets. In total, 60 AI-generated diet plans (4 profiles × 5 AI models × 3 days) were produced and evaluated. In addition, four one-day dietitian-designed reference diets (one per profile) were created and used solely as fixed comparators. Thus, the total number of diet plans generated was 64, while statistical analyses were conducted on the 60 AI-generated plans.

All statistical operations were performed using IBM SPSS Statistics 29.0 (IBM Corp., Armonk, NY, USA). In all analyses, the assumption of normality was evaluated using the Shapiro–Wilk test, and a single-sample *t*-test was applied when parametric conditions were met, with the dietitian-designed reference diet serving as the test value. Effect sizes were calculated using Cohen’s d and interpreted according to established thresholds: 0.20–0.49 small, 0.50–0.79 medium, and ≥0.80 large effect sizes (33). The differences between AI models and dietitian-prepared reference plans were evaluated using single-sample *t*-tests for energy and macronutrients. Bland–Altman analysis was applied to assess the level of agreement between methods, with mean difference (BIAS) and 95% limits of agreement (LOA) calculated for each nutrient. As Bland–Altman analysis assumes independent paired observations, this assumption was addressed by defining the primary unit of analysis at the aggregated AI model–profile level, using mean values derived from three-day AI-generated outputs. This approach reduced within-model dependency and enabled evaluation of systematic agreement with guideline-based reference diets. In addition, proportional bias was assessed using simple linear regression to determine whether differences varied according to nutrient magnitude. Heatmap analyses were generated to visually examine micronutrient variability across models. Statistical significance was set at *p* < 0.05 for all analyses.

## 3 Results

### 3.1 Comparison of dietitian and AI-generated diets

When the macronutrient distribution is examined as a percentage of total energy, according to the BeBIS analysis, the CHO percentages in dietitian plans are 44, 46, 45 and 44% for G-OW, G-OB, B-OW and B-OB, respectively; protein percentages 19, 18, 19, and 20%; lipid percentages were calculated as 37, 36, 36 and 37%. Although this distribution includes small fluctuations due to individual differences, it shows that the total macro profile generally fits the AMDR ranges (CHO 45–65%, lipid 25–35%, protein 10–30%) and that dietitian plans serve as a guideline-based reference. In contrast, AI-generated diets consistently exhibited higher lipid ratios (41.5–44.5%) and protein ratios (21.5–23.7%), while CHO percentages (32.4–36.3%) were markedly lower. This pattern illustrates a systematic shift across all AI models to lower CHO, higher protein, and higher lipid meal structures, indicating that the macronutrient balance, not just the amount of gram-based nutrients, is significantly disrupted in AI-generated plans (Supplementary Table S1).

A one-sample *t*-test was conducted to compare the differences between dietitian-designed and AI-generated diet plans for energy and macronutrient contents. The mean bias for total energy was 695.4 ± 388.2 kcal (*t*(59) = 13.88, *p* < 0.001), showing that AI diets provided lower total energy values. Similar trends were observed for macronutrients: protein (19.9 ± 22.6 g, *t*(59) = 6.83, *p* < 0.001), lipid (15.8 ± 22.8 g, *t*(59) = 5.38, *p* < 0.001), and CHO (114.6 ± 39.7 g, *t*(59) = 22.39, *p* < 0.001). Effect sizes were large for energy (d = 1.79), protein (d = 0.88), and CHO (d = 2.89), and moderate for lipid (d = 0.69). These results highlight substantial practical discrepancies between the two approaches (Table 1).

**Table 1:** Single-sample *t*-test results on the energy difference between dietitian and artificial intelligence diets.

Değişken	*N*	Average difference (mean ± SD)	95% confidence interval (upper-lower)	*t* (SD)	*p*	Cohen’s *d*
Energy (kcal)	60	695.38 ± 388.21	595.09–795.67	13.88 (59)	**<0.001**	1.79
Protein (g)	60	19.95 ± 22.64	14.10–25.80	6.83 (59)	**<0.001**	0.88
Lipid (g)	60	15.84 ± 22.83	9.95–21.74	5.375(59)	**<0.001**	0.69
CHO (g)	60	114.62 ± 39.66	104.37–124.86	22.39 (59)	**<0.001**	2.89

Bold values indicate statistically significant results (*p* < 0.05).

When AI models were compared with each other, significant differences were observed in total energy (Kruskal–Wallis H = 17.68, *p* = 0.001), lipid (H = 24.85, *p* < 0.001), and carbohydrate content (H = 15.76, *p* = 0.003). In contrast, no significant difference was found among AI models with respect to protein content (H = 3.29, *p* = 0.510). These findings indicate that AI models exhibit model-dependent variability in energy, fat, and carbohydrate generation, whereas protein distribution appears to be relatively consistent across models. Model-specific median and interquartile range values for energy and macronutrients are presented in Supplementary Table S2.

### 3.2 Limits of agreement analysis (Bland–Altman plots)

**Figure 2:**
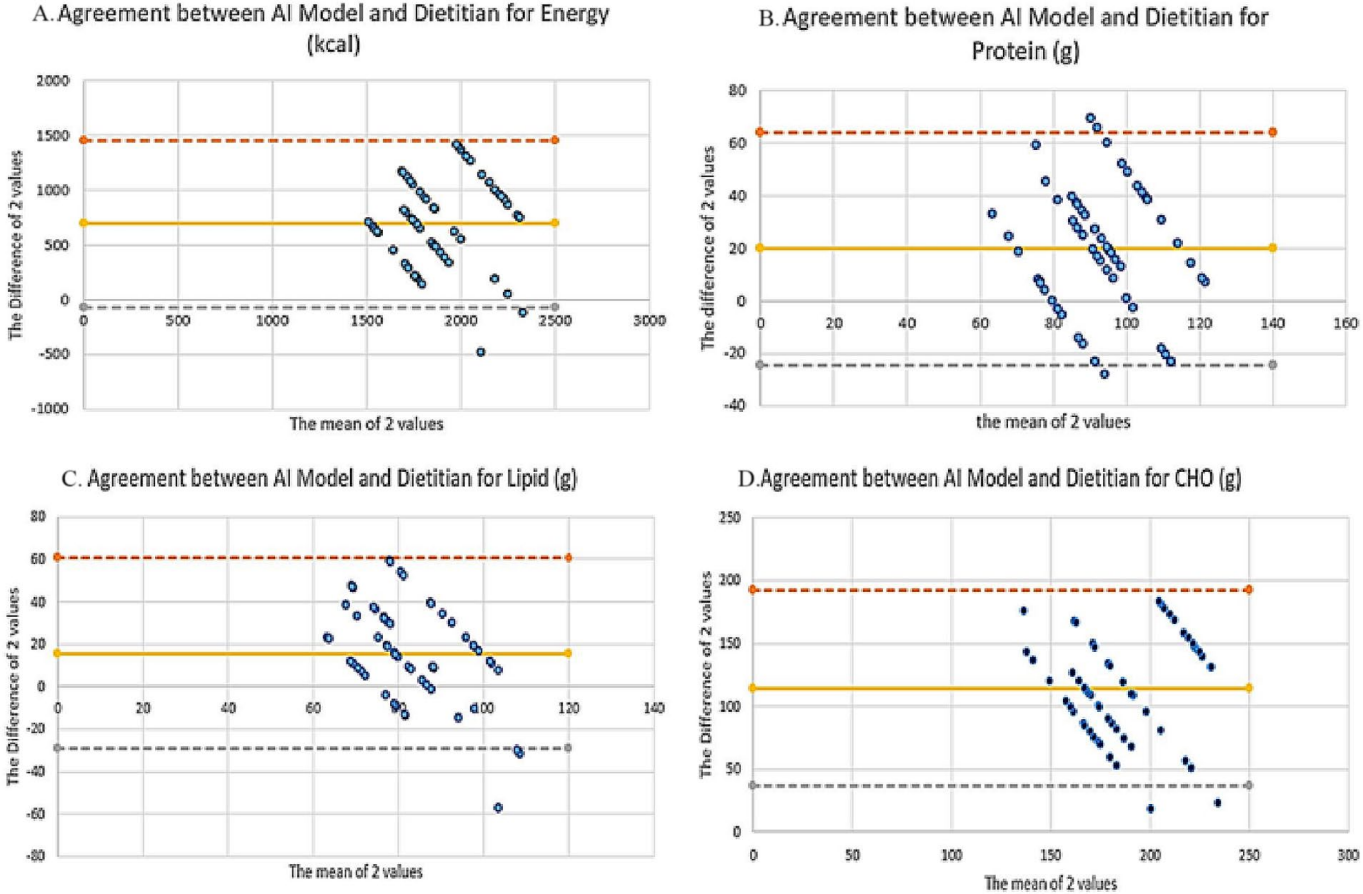
Bland–Altman plots comparing dietitian-designed and AI-generated diet plans for energy and macronutrients. Panels show the agreement between dietitian and AI-generated values for **(A)** energy (kcal), **(B)** protein (g), **(C)** lipid (g), and **(D)** carbohydrate (g). Differences were calculated as “Dietitian − AI.” The yellow solid line represents the mean bias, while the gray and orange dashed lines denote the 95% limits of agreement (LOA). A positive bias indicates that AI-generated diet plans systematically underestimated nutrient values compared with the dietitian reference (created using Microsoft Excel).

Bland–Altman plots were generated to assess the agreement between AI and dietitian results (Figure 2). For energy, the mean bias was +695.4 kcal, with 95% limits of agreement (LOA) ranging from −81.8 to +1472.6 kcal, suggesting a systematic underestimation of total energy by AI models. Across energy and macronutrients, the proportion of observations falling outside the 95% limits of agreement ranged from 3.3 to 5.0%, indicating limited extreme disagreement. Detailed numerical outputs for Bland–Altman analysis, including proportional bias testing, are presented in Supplementary Table S3. The proportion of observations falling outside the 95% limits of agreement for energy and macronutrients is presented in Supplementary Table S4.

For macronutrients, the bias and LOA values were as follows:

- Protein: +19.9 g (95% LOA: −24.4 to +64.3 g).
- Lipid: +15.8 g (95% LOA: −28.9 to +60.6 g).
- CHO: +114.6 g (95% LOA: +36.9 to +192.3 g).

In all plots, the majority of data points were positioned above the zero line, indicating that AI-generated diets consistently yielded lower macronutrient and energy estimates compared to the dietitian reference.

### 3.3 Regression analysis for proportional bias

Simple linear regression analyses were used to determine whether proportional bias was present, with mean nutrient values serving as predictors of the differences between methods. No significant proportional bias was observed for energy (*R*^2^ = 0.001, *p* = 0.777), protein (*R*^2^ = 0.012, *p* = 0.400), or CHO (*R*^2^ = 0.002, *p* = 0.718). However, a significant negative association was detected for lipid (*R*^2^ = 0.220, *p* < 0.001; B = −0.931), suggesting that discrepancies decreased as lipid levels increased. This indicates the presence of proportional bias for lipid content only (Table 2).

**Table 2:** Results on Bland–Altman regression analysis for energy and macronutrients.

Variable	*B*	Std. error	*t*	*p*	*R*	*R* ^2^	*F*_(1, 58)_	Model *p*	Comment
Constant	573.17	432.60	1.325	0.190	–	–	–	–	–
Energy mean (kcal)	0.065	0.228	0.284	0.777	0.037	0.001	0.081	0.777	No proportional bias
Protein mean (g)	−0.195	0.230	−0.848	0.400	0.111	0.012	0.719	0.400	No proportional bias
Lipid mean (g)	−0.931	0.230	−4.050	**<0.001**	0.470	0.220	16.404	<0.001	Proportional bias present
CHO mean (g)	0.074	0.203	0.363	0.718	0.048	0.002	0.131	0.718	No proportional bias

* *p* < 0.05, regression analysis. Bold values indicate statistically significant results (*p* < 0.05).

### 3.4 Micronutrient comparison

**Figure 3:**
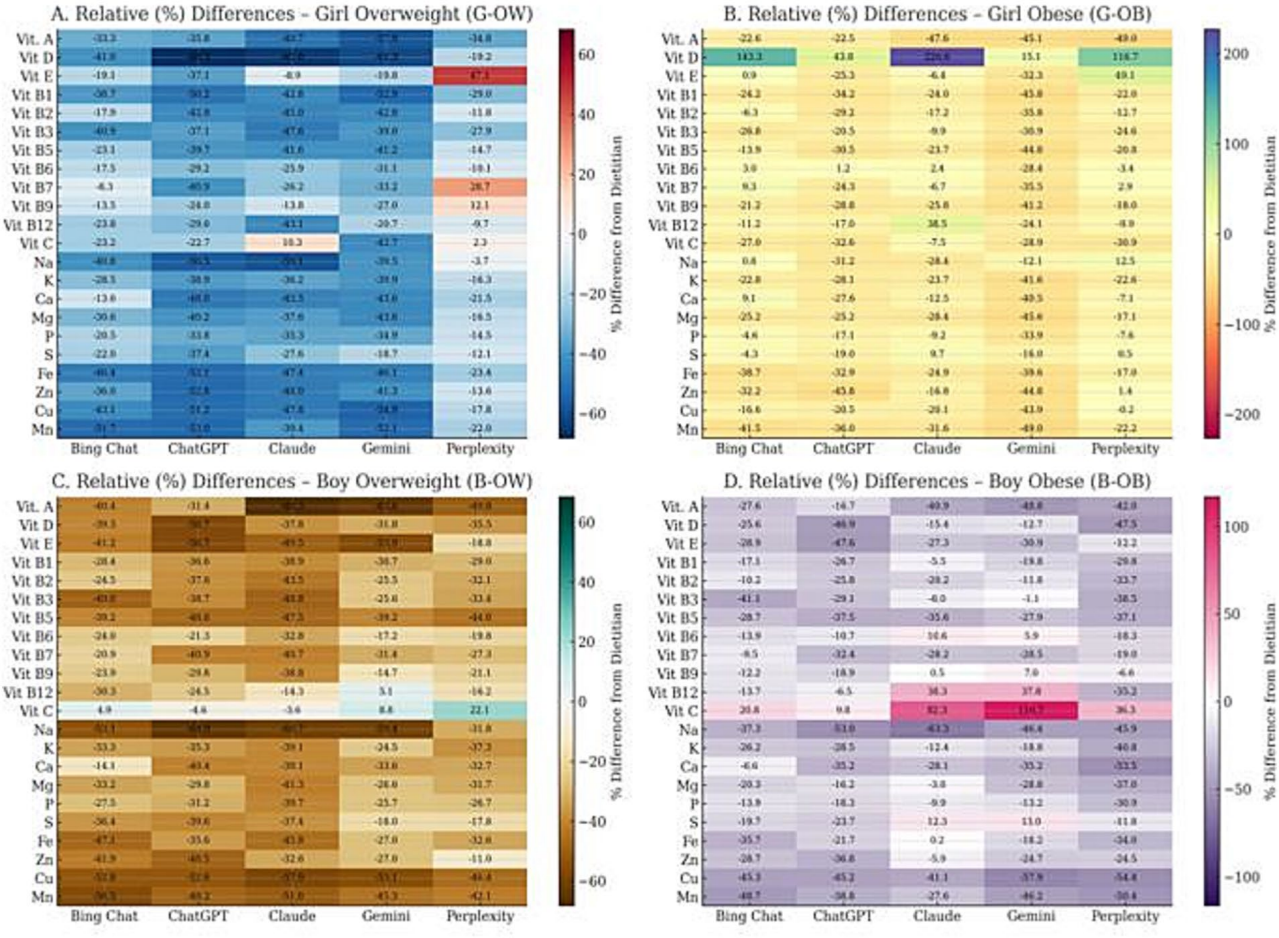
Comparison of micronutrient distributions among dietitian and AI-generated diet plans across subgroups. **(A)** Girl–Overweight, **(B)** Girl–Obese, **(C)** Boy–Overweight, **(D)** Boy–Obese. The heatmap visualizes the mean values of 22 micronutrients estimated by five large language models (ChatGPT, Gemini, Claude, Bing Chat, and Perplexity) compared to the dietitian-designed reference diets. Color intensity represents relative nutrient content differences, where lighter shades indicate values closer to the dietitian reference, and darker tones indicate higher deviations across AI models (created using Python (Matplotlib, Seaborn)).

Heatmap visualizations (Figure 3) illustrated the distribution of 22 micronutrients across AI-generated and dietitian-designed diet plans. Distinct trends were observed between demographic subgroups and AI models.

In girl–overweight and girl–obese profiles, discrepancies were most pronounced for vitamin D, folate, and magnesium, where Bing Chat and Perplexity tended to overestimate values. Conversely, ChatGPT and Claude yielded estimates closer to the dietitian benchmark.

In boy–overweight and boy–obese subgroups, greater divergences emerged for vitamin C, calcium, and phosphorus, with ChatGPT and Gemini producing higher-than-reference estimates. Across all profiles, vitamins B_1_, B_2_, B_6_, and B_12_ demonstrated strong consistency between AI and dietitian outputs, while trace minerals (iron, zinc, copper, manganese) varied markedly across models.

Collectively, these findings suggest that AI models can approximate general micronutrient patterns but remain inconsistent in certain nutrient categories. Therefore, AI-generated diet plans should be interpreted with professional oversight, particularly where micronutrient precision is critical.

## 4 Discussion

The use of AI-supported systems in healthcare has been the subject of increasing research in recent years; The reliability and clinical applicability of these technologies are discussed, especially in specialized areas such as personalized nutrition planning. This study presents a comparative analysis of diet lists created by different AI models with dietitian-planned reference patterns for adolescents and comprehensively reveals the extent to which AI-based nutrition plans are reliable in terms of macro and micronutrients.

This study makes a unique contribution in that it provides a comprehensive analysis in which five different large language models (LLMs) are compared on the basis of nutritional needs specific to the adolescent population for the first time in the literature, and multi-day menu outputs are evaluated on both macronutrients and 22 micronutrients. Previous research has mostly focused on a single AI model, adult populations (34–36). In this respect, this study fills an important gap in the literature by providing a methodologically strong, systematic, and multidimensional evaluation of the safety of AI in pediatric and adolescent nutrition.

In this study, it was found that the AI models calculated the energy requirement on average −695.4 ± 388.2 kcal lower compared to the dietitian plan (*p* < 0.001; see Table 1). The effect size obtained for this difference (d = 1.79) is large enough to have serious clinical consequences in practice. This was confirmed by Bland–Altman analyses, which showed that the range of 95% LOA: −81.8 to +1472.6 kcal for energy was extremely wide. These wide ranges suggest that consistency in energy estimates of AI-generated plans is low, and clinical validity is at risk (Figure 2). There are similar findings in the literature; Özlü Karahan and Kenger (34) reported that ChatGPT-4o deviated from target energy by 10–22% across different dietary models. Another recent study examined the capacity to create personalized diets for hypothetical patients with obesity, CVD, and T2DM in terms of energy intake, nutrient accuracy, and meal variety, with recommended daily calorie intakes deviating from target energy levels by up to 20% (35).

The findings obtained in this study reveal a more critical picture when the high energy requirements of adolescents are considered. In this study, the target energy level was not clearly stated in the claim. In the study of Papastratis et al., a significant decrease in calorie deviations was reported when the energy level was given (35). However, another study by Niszczota and Rybicka (36) found that even when ChatGPT was given clear energy levels, the model was unable to adapt the total energy content of menus to these goals and made energy calculation errors at some meals. Therefore, even if the model is told to “generate recommendations in accordance with scientific guidelines”, current evidence suggests that AI models may be technically inadequate to accurately reflect quantitative components such as energy and macronutrient calculations. Failure to systematically meet energy requirements may have negative consequences on growth, metabolic balance and cognitive development in adolescents (37).

When the macronutrient composition of the diet plans created by AI models is examined, it is seen that the deviations detected in our study significantly coincide with the trends reported in the literature. According to our findings, the amount of CHO s in AI plans was significantly lower (−114.6 ± 39.7 g) compared to the reference plans prepared by the dietitian, and it was determined that only about 32.4–36.3% of the energy was provided from CHO s (Table 1; Figure 2D). This rate remains well below the 45–65% AMDR limits for adolescents defined by the IOM. The possible mechanism of this pattern could be that AIs’ training data is overly influenced by weight-loss-focused low-carb or ketogenic dietary patterns that are dominant online.

Low-CHO intake causes daily fiber consumption to decrease to insufficient levels such as 14–16 g (38). Although there are few studies, studies conducted with young people have shown no difference in weight loss between calorie-restricted low-fat and low- CHO diets and have revealed that the most important factor for weight loss is energy restriction, regardless of macronutrient distribution (39, 40). Therefore, the long-term safety of low-CHO diets in adolescence remains unclear and caution is recommended (41). According to NHANES 2017–2018 data, the average self-reported dietary intake of children and adolescents aged 2–19 years was determined as 14 g/day, which is significantly lower than the recommended level of at least 26 g in children aged 9 years and older (42). It has been reported that low fiber intake is one of the most frequently associated dietary factors with functional constipation in children and adolescents and that fiber plays a fundamental role in stool volume, intestinal transit time and composition of the intestinal microbiota (43). It is also stated that insufficient fiber consumption is associated with deterioration in intestinal habits and decrease in microbial diversity in the pediatric population, and that fiber deficiency may adversely affect intestinal health through short-chain fatty acid production (44).

In our study, protein content was examined, an average increase of +19.9 g was observed in AI blueprints, resulting in approximately 21.5–23.7% of the energy coming from protein (Table 1). This rate is above the USDA-recommended range of 10–20% (45). It has been reported that high protein intake in adolescents is associated with increased urea production and renal workload, and urinary calcium excretion is also increased (46). In addition, protein-based diets have been shown to reduce overall nutrient diversity by narrowing energy distribution and are associated with lower diet diversity scores in adolescents (47). The systematic low-planning of CHO content by AI models has been reported in the literature, and it is stated that ChatGPT and similar models reflect popular low- CHO or ketogenic diet trends, especially in the context of weight management (48). This trend is more evident in the study by Özlü Karahan and Kenger, in which they examined the performance of ChatGPT-4o in generating sample menus for weight management purposes. In the study in question, while 52% of the energy was provided from CHO s and 16% from protein in the reference menus prepared by dietitians, the CHO ratio reached only 23 ≈ % and the protein ratio reached 27–28% in the menus produced by ChatGPT-4o. In addition, the fiber content in AI menus was found to be significantly lower compared to reference plans; the energy content deviated systematically (34). These findings strongly align with the pattern of low CHO (≈38%), high protein (23–25%), and high lipid (37–38%) observed in the diets produced by AI models in our study. The two studies have shown similar results, particularly in terms of CHO and fiber deficiency, suggesting that AI models tend to diverge from scientific guidelines, mirroring popular low-CHO eating approaches.

Findings on lipid intake similarly show that AI models exceed recommended ranges. In this study, it was found that lipid energy increased to an average of 41.5–44.5% in AI plans (Table 1; Figure 2C), which is above the USDA’s recommendations of 25–30% (45). It is reported that low adherence to dietary guidelines in adolescents is associated with higher cardiometabolic risk and adiposity levels (49). Similarly, high variation in the fatty acid distribution of diet plans created by different AIs and out-of-grid values have been reported, which has been found to be clinically inconvenient, especially in groups at risk of chronic diseases (16). In our study, Bland–Altman regression analysis for lipid content revealed a statistically significant proportional bias (*R*^2^ = 0.22; *p* < 0.001), indicating that discrepancies between AI-generated diets and reference plans decreased as lipid values increased, while greater variability was observed at low to moderate lipid levels (Figure 2B). The observed reduction in differences at higher mean values may be partially explained by regression toward the mean and the tendency of AI models to rely on fixed portion templates when generating higher-lipid meal plans. This pattern suggests that AI outputs may be influenced by commonly encountered dietary patterns in publicly available data sources rather than strictly adhering to evidence-based nutritional guidelines.

When evaluated in terms of micronutrients, both low levels and inconsistencies ranging from model to model were observed in the diet plans created by the AI models, especially in nutrients such as vitamin D, folate, calcium, iron, and magnesium (Figure 3). These findings should be carefully evaluated, especially when considering the clinical significance of micronutrient adequacy in growing individuals. These results are in line with studies in the literature that reveal limitations in micronutrient predictions of AI-based systems. Naja et al. (50) highlighted that chatbot-based nutrition apps used in metabolic syndrome and diabetes management exhibit varying levels of accuracy regarding micronutrient content, with some models capable of producing values below or above reference requirements. Bayram and Arslan’s (51) study revealed distinct inconsistencies and inefficiencies in ChatGPT-generated diet plans, particularly in micronutrients such as calcium, iron, and vitamin D. In the results of the study, they reported that careful evaluation should be made especially in elements such as vitamin D, folate and potassium (51). The significant pattern differences in vitamin D and folate levels in this study are consistent with these findings.

This wide variability in micronutrients suggests that AI-generated diets can lead to additional deficiencies, especially in adolescents who are already at risk of deficiencies in vitamin D, calcium, iron, and folate. Therefore, it shows that it is inconvenient to transfer such plans directly to clinical practice.

It has been defined in the literature that long-term overly restrictive eating patterns carry significant risks for the onset of eating disorders in adolescence (52). Body dissatisfaction accompanying restrictive diet practices brings risks such as inadequate food intake in children and adolescents, excessive weight gain due to binge eating episodes that occur after food restriction, and the implementation of harmful weight control behaviors (53). *Adolescents may be particularly susceptible to misleading or oversimplified health information presented through digital platforms.* In addition, there are significant limitations to the clinical accuracy of AI-based models. One of the significant limitations of AI models when generating nutritional recommendations is their *tendency to generate hyper-adaptive responses aimed at satisfying the user.* This makes the model more likely to provide answers that are shaped by the user’s expectations, putting scientific accuracy on the back burner. When all these features are considered together, it is clear that algorithm-based nutritional recommendations may carry potential risks for adolescents.

Overall, the wide deviations observed in macro- and micronutrient content between AI-generated diets and guideline-based reference plans may have adverse implications for growth, bone mineralization, cognitive development, and metabolic health during adolescence. These findings indicate that AI-based diet plans should not be used as standalone tools in this population and should only be considered under professional supervision and within a clinical context.

When considered collectively, the findings of this study contribute to a more comprehensive understanding of both intra-model and inter-model variability in AI-based nutrition planning, helping to identify areas in which such tools may be cautiously applied as well as aspects that may pose clinical risks in adolescent populations. In this regard, while AI models may offer rapid and accessible support in nutrition-related applications, they should be regarded as complementary tools rather than substitutes for dietitians in the medical nutrition therapy of adolescent obesity.

Role-based prompts instructing AI models to act as registered dietitians were intentionally omitted, as the primary aim was to reflect typical user–AI interactions without a professional framework. Such prompts may artificially enhance output quality and reduce ecological validity; therefore, their potential impact on nutritional accuracy should be explored in future studies.

This study has several strengths. Firstly, the fact that five different AI models were evaluated with standardized prompts in the same way significantly increased the comparison power and methodological consistency between models. In addition, the creation of a three-day diet plan for each profile allowed for the analysis of intra-model reproducibility, consistency and variation, providing a more robust evaluation compared to previous literature. The fact that the reference plans prepared by the dietitian were based on international scientific guidelines such as WHO, FAO and TÜBER guidelines and AMDR provided a reliable clinical reference point for comparisons; The comprehensive evaluation covering energy, macro and 22 micronutrients strengthened the analytical depth of the study.

## 5 Limitations

Since the study design was based on simulated scenarios, real-life nutritional behaviors, adaptation processes, and metabolic responses of adolescents could not be evaluated. Given the rapidly evolving nature of AI models, the findings reflect only the specific versions tested and may not be generalizable to future iterations. Although four standardized adolescent profiles were included, variations in age, physical activity levels, socioeconomic background, and comorbid conditions were not examined. Additionally, the use of Turkish-language prompts may limit the generalizability of the findings to other linguistic and cultural contexts. Although AI-generated diet plans were produced across repeated profiles and models, the primary unit of analysis was defined at the aggregated model–profile level by averaging three-day outputs. This approach was intentionally selected to reduce within-model variability and to focus on systematic deviation from guideline-based reference diets. Nevertheless, the repeated and hierarchical structure of the data may limit full statistical independence, and this should be considered when interpreting the results. Similarly, Bland–Altman analysis assumes independent paired observations; therefore, limits of agreement should be interpreted with caution in light of the aggregated analytical approach. Future studies incorporating mixed-effects modeling or real-world individual-level data may provide more refined estimates of variability and agreement.

This study did not incorporate prior dietary intake records, as such data would require assumptions regarding recall accuracy and eating behavior, potentially introducing additional bias into a simulation-based design. Given these limitations, further studies supported by real-life data and prospective designs are warranted to validate and extend the present findings.

## Data Availability

The original contributions presented in the study are included in the article/Supplementary material, further inquiries can be directed to the corresponding author.

## References

[1] World Health Organization. Obesity and overweight. Geneva: WHO (2024).

[2] UNICEF. The state of the world’s children 2019: Children, food and nutrition – Growing well in a changing world. New York: UNICEF (2019).

[3] Kansra AR Lakkunarajah S Jay MS. Childhood and adolescent obesity: a review. Front Pediatr. (2021) 8:581461. doi: 10.3389/fped.2020.581461, 33511092 PMC7835259

[4] Curis C Ardeleanu V Moroianu LA Manole C Stoica RA Gherghiceanu F . Obesity in children: systematic review over a 6-year period. J Mind Med Sci. (2024) 11:310–20. doi: 10.22543/2392-7674.1543

[5] Jalali-Farahani S Alamdari S Karimi M Amiri P. Is overweight associated with health-related quality of life (HRQoL) among Tehranian school children? SpringerPlus. (2016) 5:313. doi: 10.1186/s40064-016-1930-1 27066345 PMC4786555

[6] World Health Organization. Noncommunicable diseases: Childhood overweight and obesity. Geneva: WHO; 2025. Available online at: https://www.who.int/news-room/questions-and-answers/item/noncommunicable-diseases-childhood-overweight-and-obesity (accessed May 20, 2025).

[7] Hampl SE Hassink SG Skinner AC Armstrong SC Barlow SE Bolling CF . Clinical practice guideline for the evaluation and treatment of children and adolescents with obesity. Pediatrics. (2023) 151:e2022060640. doi: 10.1542/peds.2022-060640, 36622115

[8] Styne DM Arslanian SA Connor EL Farooqi IS Murad MH Silverstein JH . Pediatric obesity—assessment, treatment, and prevention: an Endocrine Society clinical practice guideline. J Clin Endocrinol Metab. (2017) 102:709–57. doi: 10.1210/jc.2016-2573, 28359099 PMC6283429

[9] Pfeifflé S Pellegrino F Kruseman M Pijollet C Volery M Soguel L . Current recommendations for nutritional management of overweight and obesity in children and adolescents: a structured framework. Nutrients. (2019) 11:362. doi: 10.3390/nu11020362, 30744122 PMC6412470

[10] Han R Todd A Wardak S Partridge SR Raeside R. Feasibility and acceptability of chatbots for nutrition and physical activity health promotion among adolescents: systematic scoping review with adolescent consultation. JMIR Hum Factors. (2023) 10:e43227. doi: 10.2196/43227, 37145858 PMC10199392

[11] Stoyanova R Shopov D Indjian K Dimova R. ChatGPT and high school students’ eating habits: benefits, risks, and insights from a cross-sectional study. Eurasia Proc Sci Technol Eng Math. (2024) 27:130–5. doi: 10.55549/epstem.1518439

[12] Liao L-L Chang L-C Lai I-J. Assessing the quality of ChatGPT’s dietary advice for college students from dietitians’ perspectives. Nutrients. (2024) 16:1939. doi: 10.3390/nu16121939, 38931294 PMC11206595

[13] Onay T Bekar D Çoban E Doğan N Günşen U. Artificial intelligence in clinical nutrition: a descriptive comparison of ChatGPT- and dietitian-planned diets for chronic disease scenarios. J Hum Nutr Diet. (2025) 38:e70135. doi: 10.1111/jhn.70135, 41054023

[14] Güner E Ülker MT. Can artificial intelligence replace dietitians? A conversation with ChatGPT. Toros Univ J Food Nutr Gastron. (2024) 3:49–56. doi: 10.58625/jfng-2474

[15] Ponzo V Rosato R Scigliano MC Onida M Cossai S De Vecchi M . Comparison of the accuracy, completeness, reproducibility, and consistency of different AI chatbots in providing nutritional advice: an exploratory study. J Clin Med. (2024) 13:7810. doi: 10.3390/jcm13247810, 39768733 PMC11677083

[16] Kaya Kaçar H Kaçar ÖF Avery A. Diet quality and caloric accuracy in AI-generated diet plans: a comparative study across chatbots. Nutrients. (2025) 17:206. doi: 10.3390/nu17020206, 39861336 PMC11768065

[17] Guo P Liu G Xiang X An R. From AI to the table: a systematic review of ChatGPT’s potential and performance in meal planning and dietary recommendations. Dietetics. (2025) 4:7. doi: 10.3390/dietetics4010007

[18] Belkhouribchia J Pen JJ. Large language models in clinical nutrition: an overview of its applications, capabilities, limitations, and potential future prospects. Front Nutr. (2025) 12:1635682. doi: 10.3389/fnut.2025.1635682, 40851903 PMC12367769

[19] ChatGPT 4.0. Open AI ChatGPT. Available online at: https://chatgpt.com/ (accessed October 15, 2025).

[20] Gemini. Google Gemini App. Available online at: https://gemini.google.com/app (accessed October 16, 2025).

[21] Bing Chat. Available online at: https://www.microsoft.com/en-us/edge/features/bing-chat?form=MT00D8 (accessed October 17, 2025).

[22] Claude Sonnet 4.5. Available online at: https://claude.ai/ (accessed October 18, 2025).

[23] Perplexity. Available online at: https://www.perplexity.ai/ (accessed October 19, 2025).

[24] World Health Organization Adolescent health WHO 2025. Available online at: https://www.who.int/teams/sexual-and-reproductive-health-and-research-(srh)/areas-of-work/adolescent-and-sexual-and-reproductive-health-and-rights (Accessed October 20, 2025).

[25] Hales C. M. Freedman D. S. Akinbami L. Wei R. Ogden C. L. 2022 Evaluation of alternative body mass index (BMI) metrics to monitor weight status in children and adolescents with extremely high BMI using CDC BMI-for-age growth charts National Center for Health Statistics. Available online at: https://www.cdc.gov/growthcharts/extended-bmi.htm (Accessed October 21, 2025). 36598420

[26] Republic of Turkey Ministry of Health. Türkiye Beslenme Rehberi (TÜBER). Ankara: Sağlık Bakanlığı Yayınları (2022).

[27] World Health Organization. (2018). Guideline: Implementing effective actions for improving adolescent nutrition. Geneva, Switzerland: World Health Organization (WHO).

[28] National Academies of Sciences, Engineering, and Medicine. Description of the acceptable macronutrient distribution range. In: Rethinking the acceptable macronutrient distribution range for the 21st century: A letter report. Washington, DC: National Academies Press (US) (2024). 39680697

[29] FAO/WHO/UNU Expert Consultation. Human energy requirements: Report of a Joint FAO/WHO/UNU Expert Consultation. Rome: Food and Agriculture Organization of the United Nations. (2001).

[30] FAO/WHO/UNU Expert Consultation. Human energy requirements. FAO F&N Tech rep 1. Rome: FAO. 200429 (2004).

[31] Türkiye Diyetisyenler Derneği [TDD], Çocuk Sağlığı ve Hastalıklarında Beslenme. Geneva, Switzerland: World Health Organization (WHO). (2018).

[32] T.C. Ministry of Health. Adolescent Healthy Nutrition and Active Living Guideline. Ankara: Ministry of Health Publications. (2012). Available online at: https://hsgm.saglik.gov.tr/depo/birimler/saglikli-beslenme-ve-hareketli-hayat-db/Dokumanlar/Kitaplar/ergenlerde_saglikli_beslenme_ve_hareketli_yasam.pdf (Accessed October 10, 2025).

[33] Cohen J. Statistical power analysis for the behavioral sciences. Hillsdale, NJ: Lawrence Erlbaum Associates (1988).

[34] Ozlu Karahan T Kenger EB. ChatGPT-4o for weight management: comparison of different diet models. Food Sci Nutr. (2025) 13:e70639. doi: 10.1002/fsn3.70639, 40678335 PMC12267882

[35] Papastratis I Stergioulas A Konstantinidis D Daras P Dimitropoulos K. Can ChatGPT provide appropriate meal plans for NCD patients? Nutrition. (2024) 121:112291. doi: 10.1016/j.nut.2023.112291, 38359704

[36] Niszczota P Rybicka I. The credibility of dietary advice formulated by ChatGPT: robo-diets for people with food allergies. Nutrition. (2023) 112:112076. doi: 10.1016/j.nut.2023.112076, 37269717

[37] Carducci B Chen ZH Campisi SC Miliku K. Adolescence as a key developmental window for nutrition promotion and cardiometabolic disease prevention. NPJ Metab Health Dis. (2025) 3:40. doi: 10.1038/s44324-025-00082-1, 41073489 PMC12514207

[38] National Academies of Sciences, Engineering, and Medicine; Health and Medicine Division; Food and Nutrition Board; Standing Committee for the Review of the Dietary Reference Intake Framework. Rethinking the acceptable macronutrient distribution range for the 21st century: A letter report. National Academies Press (US). 2024. Available online at: http://www.ncbi.nlm.nih.gov/books/NBK610329/ (Accessed October 22, 2025). 39680697

[39] Demol S Yackobovitch-Gavan M Shalitin S Nagelberg N Gillon-Keren M Phillip M. Low-carbohydrate (low & high-fat) versus high-carbohydrate low-fat diets in the treatment of obesity in adolescents. Acta Paediatr. (2009) 98:346–51. doi: 10.1111/j.1651-2227.2008.01051.x 18826492

[40] Gow ML Ho M Burrows TL Baur LA Stewart L Hutchesson MJ . Impact of dietary macronutrient distribution on BMI and cardiometabolic outcomes in overweight and obese children and adolescents: a systematic review. Nutr Rev. (2014) 72:453–70. doi: 10.1111/nure.12111., 24920422

[41] Neyman A Hannon TS Committee On Nutrition. Low-carbohydrate diets in children and adolescents with or at risk for diabetes. Pediatrics. (2023) 152:e2023063755. doi: 10.1542/peds.2023-063755. 37718964

[42] Martin C. L. Clemens J. C. Moshfegh A. J. 2023 Beverage choices among children: What we eat in America, NHANES 2017–2018. Geneva, Switzerland: World Health Organization (WHO). 36724287

[43] Hojsak I Benninga MA Hauser B Kansu A Kelly VB Stephen AM . Benefits of dietary fibre for children in health and disease. Arch Dis Child. (2022) 107:973–9. doi: 10.1136/archdischild-2021-323571, 35277379 PMC9606532

[44] Salvatore S Battigaglia MS Murone E Dozio E Pensabene L Agosti M. Dietary fibers in healthy children and in pediatric gastrointestinal disorders: a practical guide. Nutrients. (2023) 15:2208. doi: 10.3390/nu15092208, 37432354 PMC10180776

[45] Snetselaar LG de Jesus JM DeSilva DM Stoody EE. Dietary guidelines for Americans, 2020–2025. Nutr Today. (2021) 56:287–95. doi: 10.1097/NT.0000000000000512, 34987271 PMC8713704

[46] Poortmans JR Dellalieux O. Do regular high protein diets have potential health risks on kidney function in athletes? Int J Sport Nutr Exerc Metab. (2000) 10:28–38. doi: 10.1123/ijsnem.10.1.28, 10722779

[47] Birch L Savage JS Ventura A. Influences on the development of children’s eating behaviours: from infancy to adolescence. Can J Diet Pract Res. (2007) 68:1–56. PMC2678872 19430591

[48] Kim DW Park JS Sharma K Velazquez A Li L Ostrominski JW . Qualitative evaluation of artificial intelligence-generated weight management diet plans. Front Nutr. (2024) 11:1374834. doi: 10.3389/fnut.2024.1374834, 38577160 PMC10991711

[49] Hu K Button AM Tate CM Kracht CL Champagne CM Staiano AE. Adolescent diet quality, Cardiometabolic risk, and adiposity: a prospective cohort. J Nutr Educ Behav. (2023) 55:851–60. doi: 10.1016/j.jneb.2023.10.003, 37897452 PMC10842960

[50] Naja F Taktouk M Matbouli D Khaleel S Maher A Uzun B . Artificial intelligence chatbots for the nutrition management of diabetes and the metabolic syndrome. Eur J Clin Nutr. (2024) 78:887–96. doi: 10.1038/s41430-024-01476-y. 39060542

[51] Bayram HM Arslan S. Nutritional analysis of AI-generated diet plans based on popular online diet trends. J Food Compos Anal. (2025) 145:107850. doi: 10.1016/j.jfca.2025.107850

[52] Nagata JM Memon Z Huang O Moreno MA. Adolescent health and generative AI—risks and benefits. JAMA Pediatr. (2026) 180:7–8. doi: 10.1001/jamapediatrics.2025.4502 41212568 PMC12621494

[53] Obeid N Flament MF Buchholz A Henderson KA Schubert N Tasca G . Examining shared pathways for eating disorders and obesity in a community sample of adolescents: the REAL study. Front Psychol. (2022) 13:13. doi: 10.3389/fpsyg.2022.805596, 35432146 PMC9008728

