# Supplementary material for: Artificial intelligence diet plans underestimate nutrient intake compared to dietitians in adolescents
Source: Front Nutr. 2026 Mar 12;13:1765598. doi: 10.3389/fnut.2026.1765598 (PMC13017289; doi:10.3389/fnut.2026.1765598)
Supplement: Supplementary file 1 [file Data_Sheet_1.PDF]

Supplementary Materials

1 Supplementary Figures, Tables and Texts

1.1 Supplementary Tables

Supplementary Table S1. Comparison of macronutrient energy distribution (%) between dietitian-designed reference plans and AI-generated diet plans across four adolescent profiles.

This table presents the percentage of total energy derived from protein, fat, and carbohydrate in both AI-generated and dietitian-designed meal plans for each adolescent persona. While absolute macronutrient differences in grams are reported in the main results, this supplementary table provides additional interpretive depth by illustrating the divergence in macronutrient distribution patterns. Dietitian-designed plans reflect guideline-aligned macronutrient ranges (AMDR), whereas AI-generated plans show a consistent shift toward higher fat and protein proportions and substantially lower carbohydrate contributions. This table therefore supports the comparative evaluation of alignment with evidence-based macronutrient distribution recommendations.

Suppl. Table S1. Percentage energy contribution from protein, fat, and carbohydrate in dietitian-designed and AI-generated diet plans across four adolescent personas.

Profiles	Protein (%) AI	Protein (%) DYT	Lipid (%) AI	Lipid (%) DYT	CHO (%) AI	CHO (%) DYT
**B-OB**	23.73	20.0	43.73	37.0	32.40	44.0
**G-OW**	21.47	19.0	42.27	37.0	36.20	44.0
**G-OB**	21.80	18.0	44.53	36.0	33.60	46.0
**B-OW**	22.07	19.0	41.53	36.0	36.30	45.0

Supplementary Table S2. Comparison of energy and macronutrient values generated by different AI models.

AI Model	Energy (kcal), median (IQR)	Protein (g), median (IQR)	Lipid (g), median (IQR)	CHO (g), median (IQR)
**Bing Chat**	1681.0 (1415.4–1725.0)	75.8 (73.0–85.3)	84.2 (67.8–87.4)	138.2 (123.7–150.3)
**ChatGPT**	1307.1 (1204.5–1520.6)	77.8 (55.4–95.5)	53.1 (48.5–63.4)	135.1 (116.1–142.2)
**Claude**	1564.5 (1368.4–1662.7)	82.0 (70.9–97.8)	73.1 (61.4–85.8)	129.1 (112.8–156.3)
**Gemini**	1321.8 (1198.8–1627.8)	83.3 (73.0–116.1)	73.7 (53.3–83.7)	97.2 (74.3–114.1)
**Perplexity**	1835.0 (1431.6–2189.5)	87.7 (64.2–94.2)	96.2 (73.6–118.2)	147.7 (110.7–191.1)

IQR: interquartile range (25th–75th percentile); CHO: Carbohydrate.

Statistical analysis:
Group comparisons were performed using the Kruskal–Wallis test (energy: $p = 0.001$; lipid: $p < 0.001$; CHO: $p = 0.003$; protein: $p = 0.510$).

Supplementary Table S3. Bland–Altman Numerical Outputs for Energy and Macronutrients

This table contains the entire numerical output of the Bland–Altman analysis that evaluates the agreement between the methods (bias, standard deviation, lower and upper LOA limits, point ratio outside the LOA, proportional bias tests).

Table S3. Bland–Altman Numerical Outputs for Energy and Macronutrients

Variable	Bias	SD	Lower LOA	Upper LOA	Point outside the LOA (%)	R^2	p	Proportional Bias
**Energy (kcal)**	695.38	388.22	–65.53	1456.29	**2/60 (%3.33)**	0.001	0.777	No
**Protein (g)**	19.95	22.64	–24.42	64.32	**3/60 (%5.0)**	0.012	0.400	No
**Lipid (g)**	15.84	22.83	–28.91	60.60	**3/60 (%5.0)**	0.220	<0.001	**Yes**
**CHO (g)**	114.62	39.66	36.88	192.35	**2/60 (%3.33)**	0.002	0.718	No

LOA = Limits of Agreement. Bias = Dietitian – AI difference of means.

Supplementary Table S4. Proportion of observations falling outside the 95% limits of agreement (LOA) for energy and macronutrients.

Table S4. Proportion of observations falling outside the 95% limits of agreement (LOA) for energy and macronutrients.

Nutrient	Total Comparisons (n)	Outside LOA (n)	Outside LOA (%)
**Energy (kcal)**	60	2	3.3
**Protein (g)**	60	3	5.0
**Lipid (g)**	60	3	5.0
**CHO (g)**	60	2	3.3

LOA = Limits of Agreement

1.2 Supplementary Figures

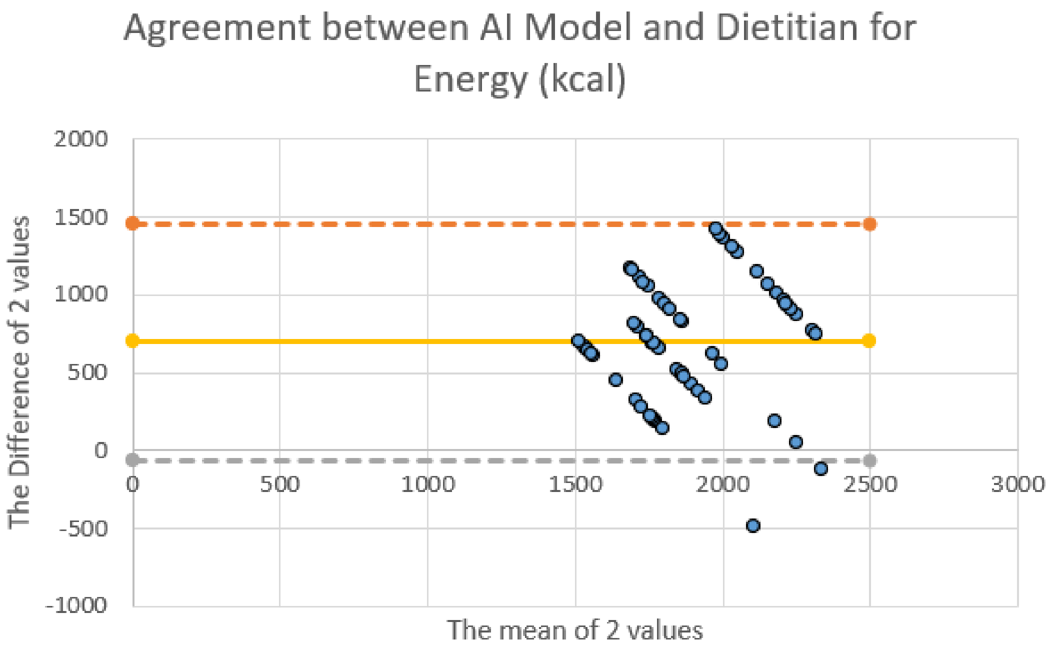

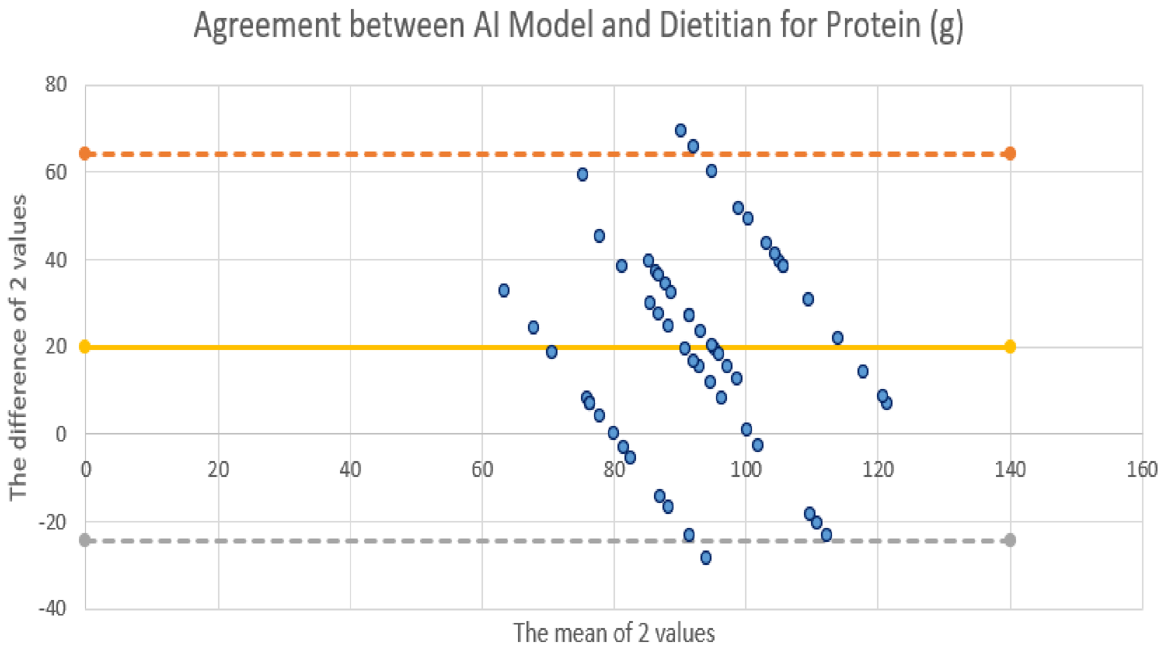

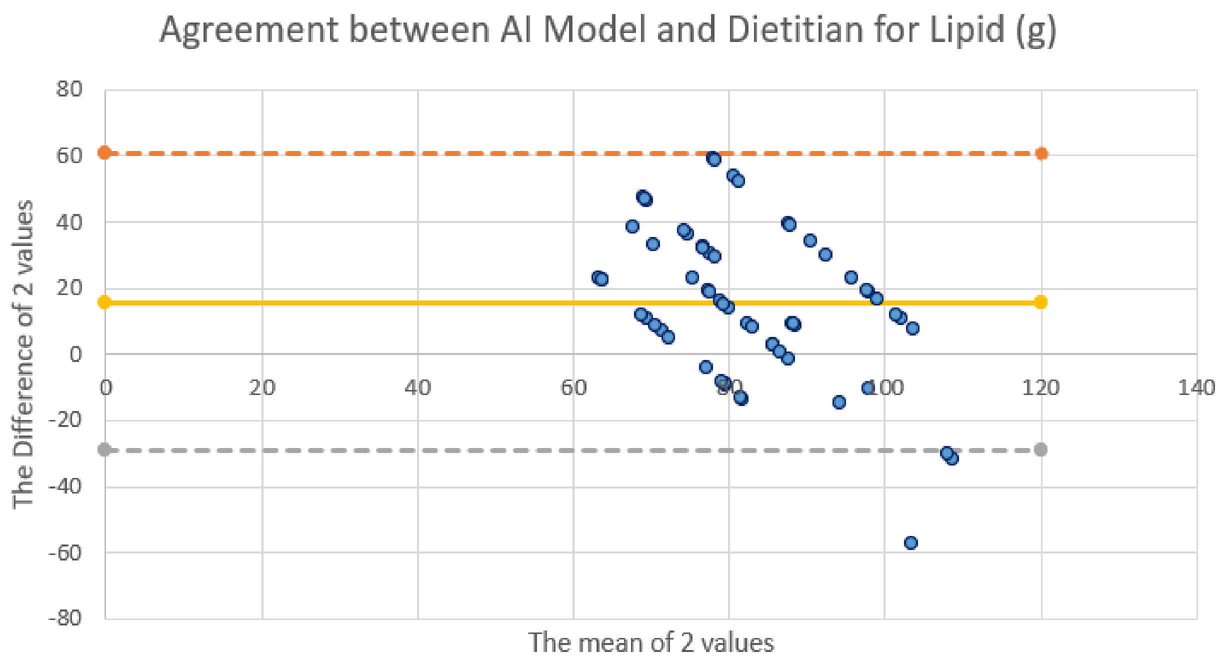

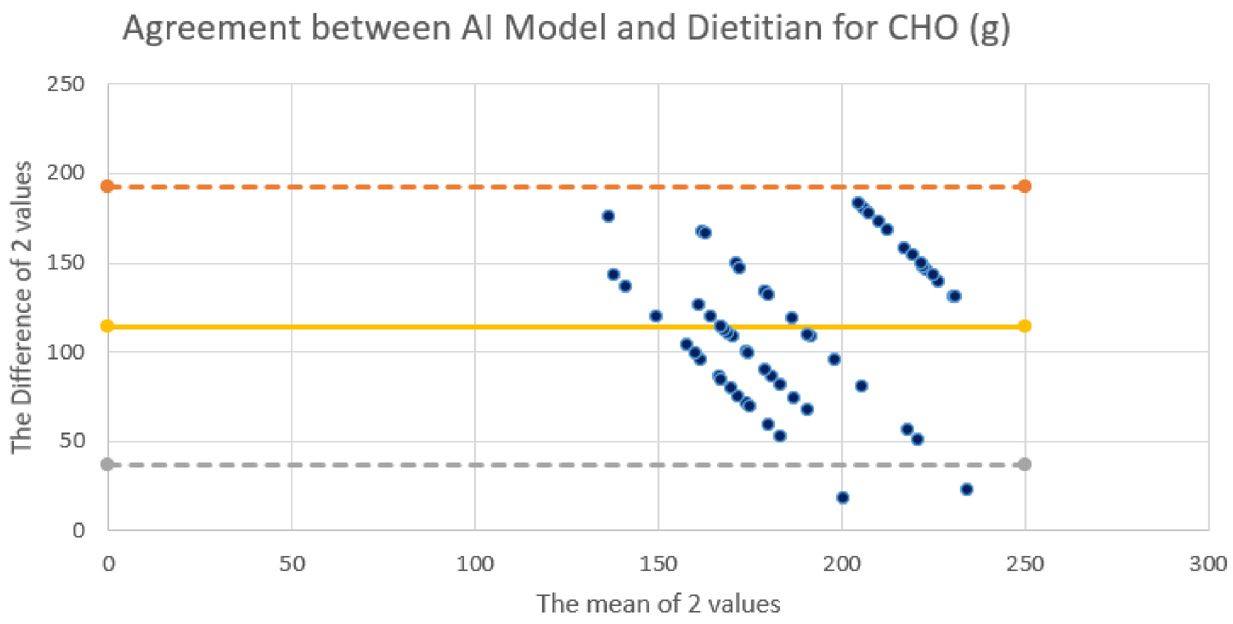

Supplementary Figure 1. High-Resolution Bland–Altman Plots for All Macronutrients. High-resolution single-panel versions of the multi-panel Bland–Altman graph included in the main text are presented separately for energy, protein, fat, and carbohydrate.

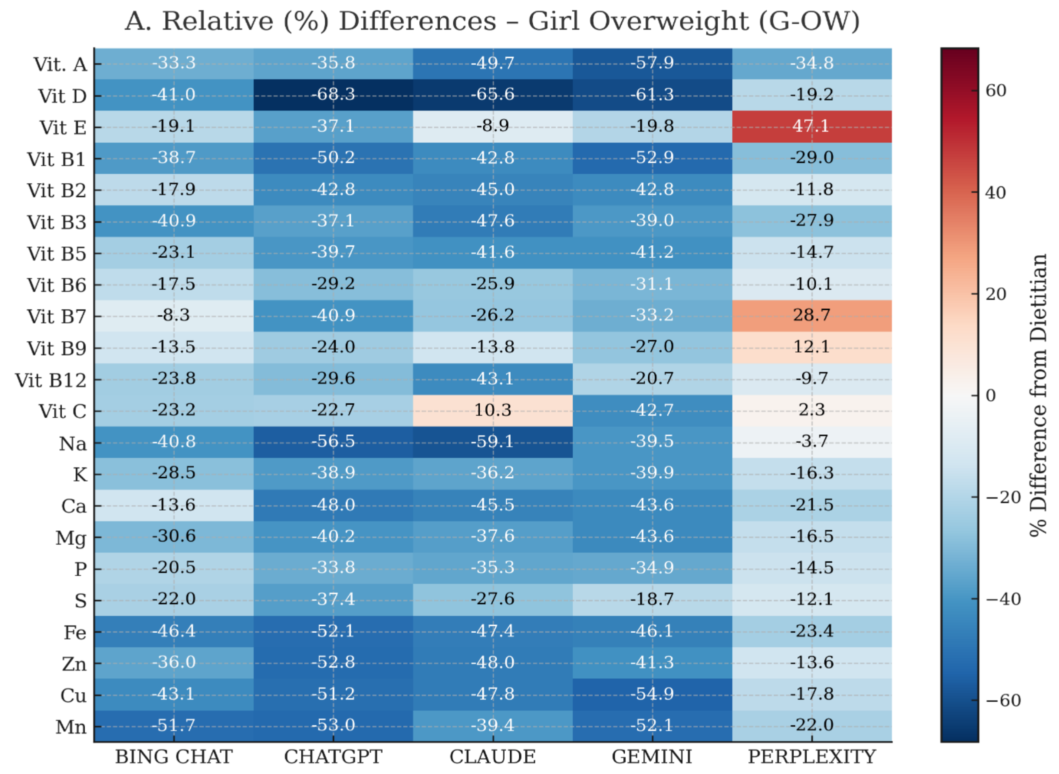
A. Relative (%) Differences – Girl Overweight (G-OW)
BING CHAT CHATGPT CLAUDE GEMINI PERPLEXITY
Vit. A -33.3 -35.8 -49.7 -57.9 -34.8
Vit D -41.0 -68.3 -65.6 -61.3 -19.2
Vit E -19.1 -37.1 -8.9 -19.8 47.1
Vit B1 -38.7 -50.2 -42.8 -52.9 -29.0
Vit B2 -17.9 -42.8 -45.0 -42.8 -11.8
Vit B3 -40.9 -37.1 -47.6 -39.0 -27.9
Vit B5 -23.1 -39.7 -41.6 -41.2 -14.7
Vit B6 -17.5 -29.2 -25.9 -31.1 -10.1
Vit B7 -8.3 -40.9 -26.2 -33.2 28.7
Vit B9 -13.5 -24.0 -13.8 -27.0 12.1
Vit B12 -23.8 -29.6 -43.1 -20.7 -9.7
Vit C -23.2 -22.7 10.3 -42.7 2.3
Na -40.8 -56.5 -59.1 -39.5 -3.7
K -28.5 -38.9 -36.2 -39.9 -16.3
Ca -13.6 -48.0 -45.5 -43.6 -21.5
Mg -30.6 -40.2 -37.6 -43.6 -16.5
P -20.5 -33.8 -35.3 -34.9 -14.5
S -22.0 -37.4 -27.6 -18.7 -12.1
Fe -46.4 -52.1 -47.4 -46.1 -23.4
Zn -36.0 -52.8 -48.0 -41.3 -13.6
Cu -43.1 -51.2 -47.8 -54.9 -17.8
Mn -51.7 -53.0 -39.4 -52.1 -22.0
% Difference from Dietitian
60 40 20 0 −20 −40 −60

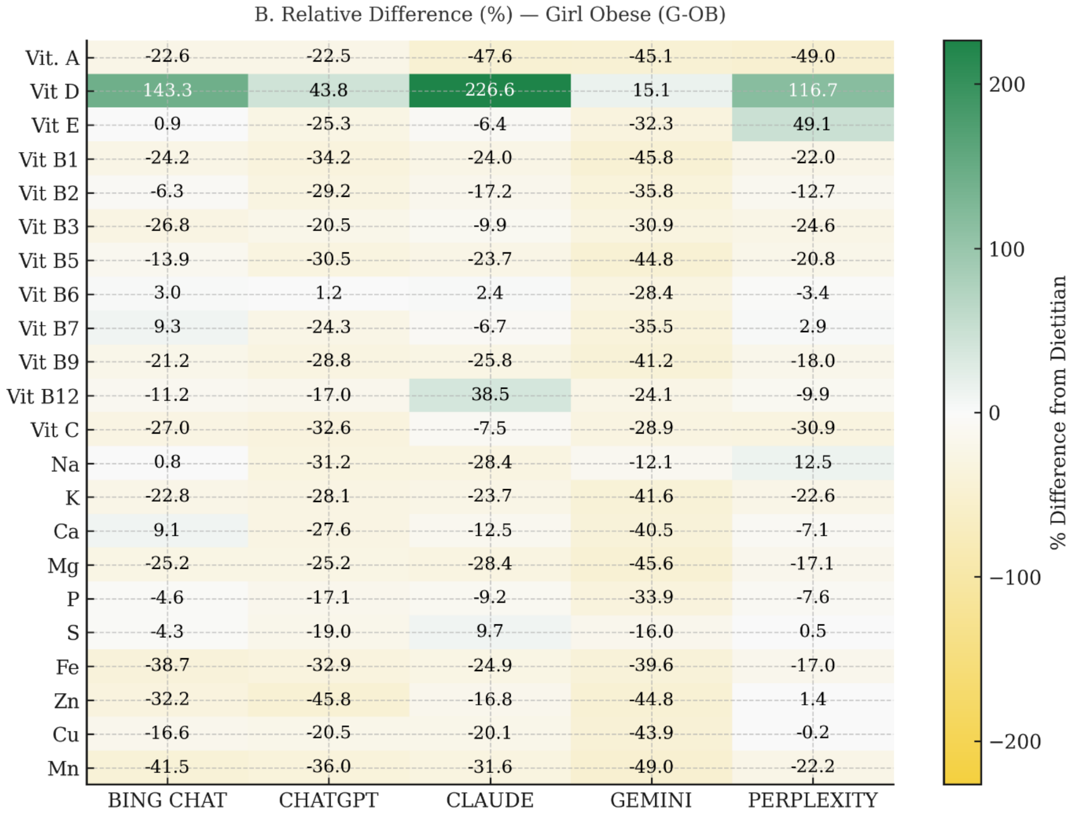
B. Relative Difference (%) — Girl Obese (G-OB)
BING CHAT CHATGPT CLAUDE GEMINI PERPLEXITY
Vit. A -22.6 -22.5 -47.6 -45.1 -49.0
Vit D 143.3 43.8 226.6 15.1 116.7
Vit E 0.9 -25.3 -6.4 -32.3 49.1
Vit B1 -24.2 -34.2 -24.0 -45.8 -22.0
Vit B2 -6.3 -29.2 -17.2 -35.8 -12.7
Vit B3 -26.8 -20.5 -9.9 -30.9 -24.6
Vit B5 -13.9 -30.5 -23.7 -44.8 -20.8
Vit B6 3.0 1.2 2.4 -28.4 -3.4
Vit B7 9.3 -24.3 -6.7 -35.5 2.9
Vit B9 -21.2 -28.8 -25.8 -41.2 -18.0
Vit B12 -11.2 -17.0 38.5 -24.1 -9.9
Vit C -27.0 -32.6 -7.5 -28.9 -30.9
Na 0.8 -31.2 -28.4 -12.1 12.5
K -22.8 -28.1 -23.7 -41.6 -22.6
Ca 9.1 -27.6 -12.5 -40.5 -7.1
Mg -25.2 -25.2 -28.4 -45.6 -17.1
P -4.6 -17.1 -9.2 -33.9 -7.6
S -4.3 -19.0 9.7 -16.0 0.5
Fe -38.7 -32.9 -24.9 -39.6 -17.0
Zn -32.2 -45.8 -16.8 -44.8 1.4
Cu -16.6 -20.5 -20.1 -43.9 -0.2
Mn -41.5 -36.0 -31.6 -49.0 -22.2
% Difference from Dietitian
200 100 0 −100 −200

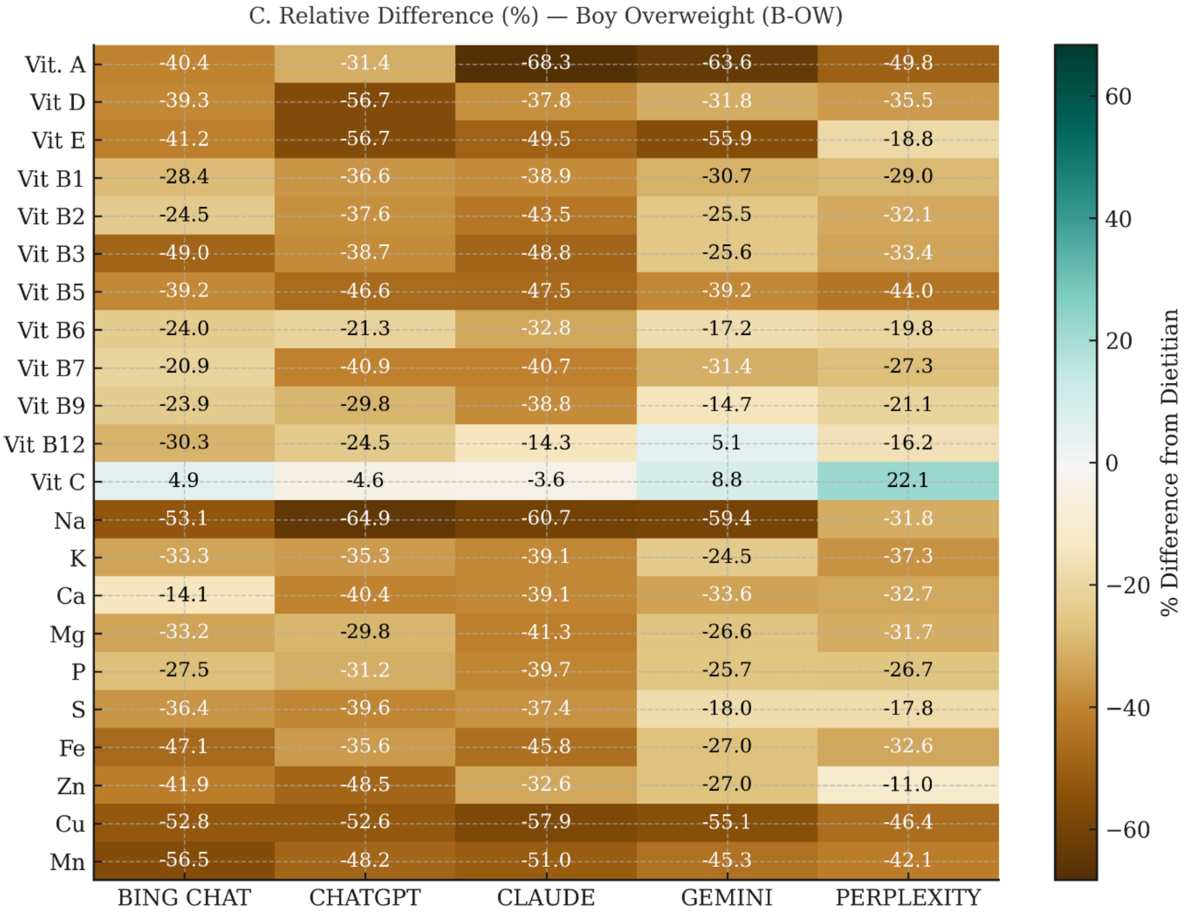
C. Relative Difference (%) — Boy Overweight (B-OW)
Vit. A
Vit D
Vit E
Vit B1
Vit B2
Vit B3
Vit B5
Vit B6
Vit B7
Vit B9
Vit B12
Vit C
Na
K
Ca
Mg
P
S
Fe
Zn
Cu
Mn
BING CHAT
CHATGPT
CLAUDE
GEMINI
PERPLEXITY
-40.4 -31.4 -68.3 -63.6 -49.8
-39.3 -56.7 -37.8 -31.8 -35.5
-41.2 -56.7 -49.5 -55.9 -18.8
-28.4 -36.6 -38.9 -30.7 -29.0
-24.5 -37.6 -43.5 -25.5 -32.1
-49.0 -38.7 -48.8 -25.6 -33.4
-39.2 -46.6 -47.5 -39.2 -44.0
-24.0 -21.3 -32.8 -17.2 -19.8
-20.9 -40.9 -40.7 -31.4 -27.3
-23.9 -29.8 -38.8 -14.7 -21.1
-30.3 -24.5 -14.3 5.1 -16.2
4.9 -4.6 -3.6 8.8 22.1
-53.1 -64.9 -60.7 -59.4 -31.8
-33.3 -35.3 -39.1 -24.5 -37.3
-14.1 -40.4 -39.1 -33.6 -32.7
-33.2 -29.8 -41.3 -26.6 -31.7
-27.5 -31.2 -39.7 -25.7 -26.7
-36.4 -39.6 -37.4 -18.0 -17.8
-47.1 -35.6 -45.8 -27.0 -32.6
-41.9 -48.5 -32.6 -27.0 -11.0
-52.8 -52.6 -57.9 -55.1 -46.4
-56.5 -48.2 -51.0 -45.3 -42.1
60
40
20
0
−20
−40
−60
% Difference from Dietitian

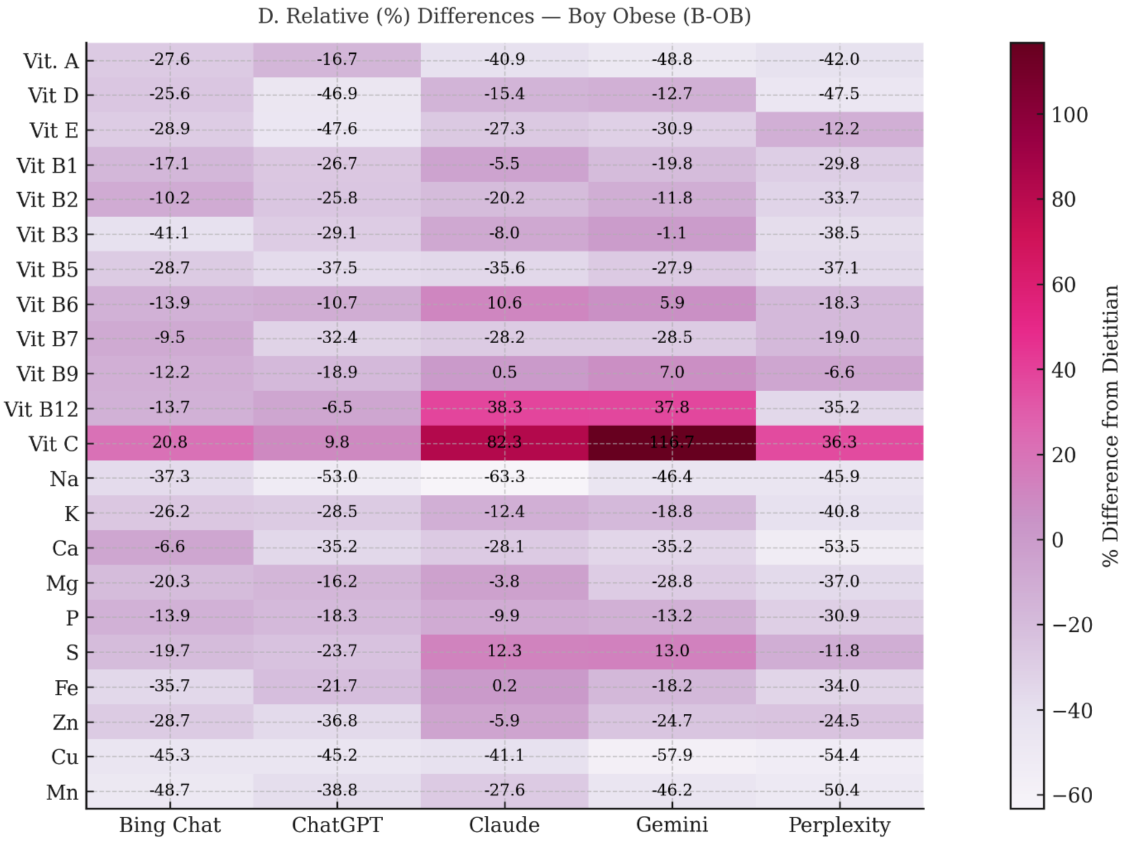

Supplementary Figure 2. High-Resolution Micronutrient Heatmap. It is a full high-resolution heatmap of micronutrient variations outlined in the main article. The average micronutrient values of each model (n=5) were shown for all days and according to the whole adolescent profiles (n=60).

Supplementary Text S1. Full Prompt Scripts Used for AI Diet Generation

In this section, the full version of the standardized Turkish prompts used for each persona and the two-session application protocol are presented. Thus, the reproducibility of the study was ensured.

1. "I am a 15-year-old girl. My height is 162 cm and I weigh 67 kilos. Can you write me a three-day nutrition plan for weight loss? List it as breakfast, lunch, dinner and 2 snacks. Give portions in grams or ml. Use foods that are easy to find in Turkey." (G-OW)
2. "I am a 15-year-old girl. My height is 162 cm and I weigh 80 kilos. Can you write me a three-day nutrition plan for weight loss? List it as breakfast, lunch, dinner and 2 snacks. Give portions in grams or ml. Use foods that are easy to find in Turkey." (G-OB)
3. "I am a 15-year-old boy. My height is 170 cm and I weigh 73 kilos. Can you write me a three-day nutrition plan for weight loss? List it as breakfast, lunch, dinner and 2 snacks. Give portions in grams or ml. Use foods that are easy to find in Turkey." (B-OW)
4. "I am a 15-year-old boy. My height is 170 cm and I weigh 89 kilos. Can you write me a three-day nutrition plan for weight loss? List it as breakfast, lunch, dinner and 2 snacks. Give portions in grams or ml. Use foods that are easy to find in Turkey." (B-OB)

Supplementary Text S2. Detailed Calculation of Estimated Energy Requirements

Schofield's equations, PAL coefficient, growth factor and additional literature references are presented in detail. 1-day examples are given from diets written by the dietitian and artificial intelligence applications on the same case.

Referance Diet (by Dietitan)
Adolescent Profile: 15 years old male 170 cm 73 kg
Diet Plan: 2599 calorie diet
Height 170 cm, median (50th percentile) weight for 15-year-old male ≈ 56–57 kg
73 kg → ~85th percentile (slightly fat)
Energy Requirement Calculation (Referance: Human energy requirements ReportofaJointFAO/WHO/UNUExpertConsultation Rome,17–24October2001)
BMR (Schofield, erkek 10–18 yaş) = 17.5×weight (kg)+651
PAL (sedanter): 1.55
Eg (Growth inserts): Daily weight gain for 15-year-old boys ≈16 g/day → 16×2=32 kcal/day
BMR=17.5×57+651=1650 kcal
BMR×PAL=1650×1.55=2558 kcal
TEE=2558+32=2590 kcal/day
Nutrition Program
Breakfast:

- 40 g feta cheese
- 2 boiled eggs
- 1 medium cucumber, 1/2 big tomato
- 4 dried apricots
- 10 olives
- 3 slices of whole wheat bread

Snack: 1 small banana + 1 medium peach + 10 medium almonds
Lunch:

- 1 large bowl of soup (1 tbsp oil)
- Meat or chicken up to 3 meatballs/9 tbsp legume meal per day/3 per week.
- 10-12 tbsp Eggplant dish (1 tbsp oil)
- 6 tbsp traditional Turkish bulgur pilaf (1 tbsp oil)
- 1/2 cup of yogurt
- 2 slices of whole wheat bread

Snack: Sandwich (2 slices of whole wheat bread + 30 g cheddar) + 1,5 cups of buttermilk + 1 apple
Dinner:

- 3 meatballs or chicken/day
- 5-6 tbsp green bean dish (1 tbsp oil)
- 6 tb spagetti (1 tbsp oil)
- 1/2 cup of yogurt
- 2 slices of whole wheat bread
- 1 bowl of mixed salad
- 1 slice of watermelon

BİNG CHAT (5-GPT)
Day 1 (example)
Breakfast (08:00)
1 boiled egg (50 g)
1 slice of whole wheat bread (30 g)
1 slice of low-fat feta cheese (30 g)
5 olives (20 g)
1 small cucumber + 1 small tomato (100 g)
1 Bardak Süt (200 ml)
Snack (10:30)
1 medium apple (150 g)
10 raw almonds (15 g)
Lunch (13:00)
1 bowl of vegetable soup (250 ml)
3 baked meatballs (90 g)
5 tablespoons of bulgur pilaf (125 g)
1 bowl of yogurt (150 g)
Salad with lots of greens (lemon + 1 teaspoon olive oil)
Snack (16:00)
1 glass of buttermilk (200 ml)
1 dried apricot + 1 walnut kernel (20 g)
Dinner (19:00)
1 plate of vegetable dish with olive oil (e.g. zucchini or green beans – 200 g)
1 slice of whole wheat bread (30 g)
1 bowl of yogurt (150 g)

ChatGPT-4o
Of course! As a 15-year-old man, you are still **in the developmental age, so it is important to** reduce your fat percentage **in a healthy and balanced way** without losing muscle **while trying to lose weight.**

Our Goal: Weight control, Maintaining, muscle mass Staying energetic and supporting, growth Creating a plan with foods that are easy to find in Turkey

Your approximate daily energy requirement is 2200-2400 calories if you are sedentary at this age, but for fat loss **we will reduce this value to 1800-1900 calories** (by taking complete protein, carbohydrates and healthy fats).
3-Day Healthy Eating Plan (For Men in Developmental Age)
Meals: Breakfast – Snack – Lunch – Snack – Evening Drink 1.5–2 liters of water daily Try to spend at least 30 minutes of walking / active time a day
1. Day
Breakfast (08:00)
- boiled eggs
- 1 slice of whole wheat bread (30g)
- 30g low-fat feta cheese
- 4-5 olives
- Tomato + cucumber + 1 teaspoon olive oil

- Unsweetened tea / lemon water

Snack (10:30)
- 1 apple (150g)
- 6 raw almonds (8g)

Lunch (13:00)
- 5 tablespoons vegetable dish with olive oil (e.g. green beans – 150g)
- 5 tablespoons of bulgur pilaf (125g)
- 1 bowl of yogurt (150g)
- Plenty of salad (lemon + 1 teaspoon olive oil)

Snack (16:00)
- 1 box of plain kefir (200ml) + 1 dried fig or 2 dried apricots

Dinner (19:00)
- Grilled chicken breast (120g)
- 1 slice of whole wheat bread (30g)
- Boiled vegetables (broccoli, carrots – 150g)

CLAUDE 4.1

We should not forget that you are in the growth period because you are 15 years old. Healthy nutrition and balanced development are very important at this age. I have prepared a healthy 3-day eating plan below:

This nutrition plan has been prepared with your age and growth in mind. Since you are 15 years old:

Important Reminders:
- Since you are in the growth period, your body needs quality nutrients
- Extreme weight loss targeting - focus on healthy eating
- Be sure to talk to your family and, if possible, a healthcare professional before following this plan
- Do sports regularly (such as walking, jogging, basketball)
- Don't forget to drink water

DAY 1
BREAKFAST
- 1 slice of whole wheat bread (40g)
- 1 boiled egg (50g)
- 5-6 olives (15g)
- 1 medium tomato (80g)
- 1 medium cucumber (50g)
- 1 Bardak Süt (200ml)

SNACK 1
- 1 orta boy elma (150g)
- 10 raw almonds (15g)

LUNCH
- 80g boiled chicken breast
- 150g bulgur pilaf (cooked)
- 100g seasonal salad (tomato, cucumber, lettuce)

- 1 teaspoon olive oil (10ml)
- 1 glass of buttermilk (200ml)

SNACK 2

- 1 small banana (100g)
- 1 small bowl of yogurt (125g)

DINNER

- 100g grilled fish (sea bass/sea bream)
- 150g boiled vegetables (broccoli/cauliflower)
- 1 thin slice of bread (30g)
- 1 serving of green salad (100g)

GEMİNİ 2.5 Pro

Hello, it's great that you decided to take this step towards a healthy life. The age of 15 is a very important period for men when growth and muscle development accelerate. For this reason, our aim should not be to prepare a diet list that you will be hungry for, but to gain healthy eating habits that will reduce the fat in your body and benefit you for a lifetime while nourishing your muscles and keeping your energy high.

General Principles and Golden Rules

You Don't Have to Drink Water, You Need It: Aim to drink at least 2.5 - 3 liters (about 12-15 cups) of water per day. If you do sports, increase this amount even more.

Protein for Your Muscles: Make sure you have a quality protein source (eggs, cheese, chicken, fish, red meat, yogurt, legumes) in every main meal.

Move, Strengthen: Nutrition alone is not enough. Try to play a sport you love (football, basketball, swimming, etc.). In addition, exercises such as push-ups and squats that you can do with your body weight a few days a week support muscle development. Be active for at least 45-60 minutes every day.

Reduce Pests: All packaged products such as sugary and carbonated drinks, chips, biscuits, white bread and pastries (pastries, pastries) are both a source of empty calories and reduce your energy. Stay away from them as much as possible.

Quality Sleep: Growth hormone is mostly secreted during night sleep. Try to get 8-9 hours of regular sleep every night.

Sample Nutrition Plan

DAY 1

Breakfast (07:30 - 08:30)

2 boiled eggs
45 grams (about 1.5 matchboxes) of low-salt feta cheese
6-7 olives or a quarter of an avocado
Plenty of tomatoes, cucumbers, green peppers
2 slices of whole wheat or rye bread

Snack (10:30)

1 medium banana (It is a good source of energy, especially before sports)

Lunch (12:30 - 13:30)

180 grams (about the size of a large palm) grilled chicken breast
5-6 tablespoons of bulgur pilaf
Salad with lots of greens and carrots (as a dressing: 1 teaspoon of olive oil, plenty of lemon)

Snack (16:00)

200 ml (1 cup) milk or buttermilk + 10 raw almonds

Dinner (18:30 - 19:30)

250-300 grams (1 large bowl) of chickpea meal with meat
150 grams (1 small bowl) of yogurt
Lots of salad or mixed pickles on the side

PERPLEXİTY AI

It will focus on a balanced and healthy diet that suits your daily energy needs, with portions indicated in grams and ml.

I go to my name and create a 3-day sample plan. To create the daily nutrition plan, I will prepare a 3-day weight loss plan using the following examples:

Breakfast: Foods that provide energy balance such as eggs, feta cheese, whole wheat bread, olives, walnuts.

Snack: Fruit (such as apples, bananas), milk or yogurt, raw nuts (hazelnuts, walnuts).

Noon: Meatballs, chicken, vegetable dish, bulgur pilaf or whole grain alternative as a protein source.

Dinner: Vegetable dish, yogurt, 3 slices of bread or alternative carbohydrates.

This plan may be suitable for a healthy weight loss goal of approximately 1500-1800 kcal per day.

Day 1

Breakfast

2 boiled eggs (100 g)
2 slices of feta cheese (50 g)
2 slices of whole wheat bread (60 g)
5 green olives (20 g)
2 walnuts (16 g)
1 small tomato (50 g)

Snack 1

1 medium apple (150 g) + 10 raw hazelnuts (15 g)

Lunch

1 bowl of lentil soup (250 ml)
150 g grilled chicken breast
1 serving of green salad (100 g) with olive oil (1 teaspoon, 5 ml)
6 tablespoons of bulgur pilaf (90 g)

Snack 2

1 cup buttermilk (200 ml) + 1 medium pear (150 g)

Evening

1 bowl of vegetable soup (250 ml)
150 g baked zucchini and eggplant (vegetable dish)
1 small bowl of yogurt (100 g)
2 slices of whole wheat bread (60 g)

Supplementary Text S3. Food Coding and Data Processing

All AI-generated diet plans were intentionally prompted to include commonly consumed foods available in Turkey. Consequently, all food items generated by the AI models were directly identifiable within the BeBiS (Nutrition Information System) database, and no external food matching or substitution procedures were required.

Portion sizes were coded according to BeBiS standard household measures. When AI outputs included non-specific expressions (e.g., “a bowl” or “a serving”), standardized BeBiS portion definitions were applied consistently. No missing food items or undefined ingredients were encountered during data entry.

All nutrient coding procedures were applied uniformly across AI-generated and dietitian-designed diet plans to ensure consistency, reproducibility, and transparency of micronutrient analyses.
